# The impact of *in utero* exposure to cancer treatments on foetal reproductive development and future fertility: a systematic review

**DOI:** 10.1093/hropen/hoaf046

**Published:** 2025-07-23

**Authors:** Elinor Sebire, Norah Spears, Rod T Mitchell, Agnes Stefansdottir

**Affiliations:** Institute of Applied Health Sciences, University of Aberdeen, Aberdeen, UK; Biomedical Sciences, University of Edinburgh, Edinburgh, UK; Centre for Reproductive Health, University of Edinburgh, Edinburgh, UK; Department of Paediatric Endocrinology and Diabetes, Royal Hospital for Children and Young People, Edinburgh, UK; Biomedical Sciences, University of Edinburgh, Edinburgh, UK

**Keywords:** fertility, chemotherapy, reproductive development, foetal outcomes, pregnancy

## Abstract

**STUDY QUESTION:**

Does cancer treatment during pregnancy affect gonadal development in the exposed foetus?

**SUMMARY ANSWER:**

Our systematic review revealed that exposure *in utero* to many cancer therapies does negatively impact gonadal development.

**WHAT IS KNOWN ALREADY:**

It is well known that many cancer therapies can have a detrimental impact on the fertility of children and young people who have been treated for cancer. However, it is not yet known how much these agents impact the gonadal development and subsequent fertility of an *in utero-*exposed foetus.

**STUDY DESIGN, SIZE, DURATION:**

We conducted a systematic review, following PRISMA guidelines, to investigate the evidence for associations between *in utero* cancer therapy exposure and gonadal development in human tissues and animal models. A systematic search was conducted across PubMed, Web of Science, and Google Scholar for titles or abstracts containing terms relating to chemotherapy or hormonal therapy agents, *in utero* exposure, and reproductive outcomes. We searched all available published articles up to July 2024.

**PARTICIPANTS/MATERIALS, SETTING, METHODS:**

Two independent reviewers performed title and abstract, then full-text screening, using inclusion/exclusion criteria decided *a priori*. We included clinical and laboratory studies on human foetal gonads and animal studies, *in vivo* and *in vitro*, where gonadal exposure occurred during the window that corresponded with human prenatal gonadal development. Data from the included studies were independently extracted and analysed by chemotherapy and hormonal drug class, focusing on reproductive outcome measures and results. Bias and quality assessments were performed with SciRAP *in vivo* or *in vitro* tool version 2.3.

**MAIN RESULTS AND THE ROLE OF CHANCE:**

3360 titles and abstracts were screened for inclusion, following the removal of duplicates, with 57 undergoing full text review and 26 eligible studies identified for inclusion (human = 4, animal-model = 22). The collated results show clear evidence of significant germ cell loss and disruption to other gonadal cell types in male and female animal-model gonadal tissues exposed both *in vivo* and *in vitro* to various chemotherapy and hormone therapies, and human male foetal tissue exposed to chemotherapy *in vitro*.

**LIMITATIONS, REASONS FOR CAUTION:**

The evidence provided was limited by the small number of studies available reporting on reproductive outcomes following *in utero* exposure to cancer therapies, a lack of comparable outcome measures, and the use of single-drug exposures compared to the more clinically relevant multi-drug combinations.

**WIDER IMPLICATIONS OF THE FINDINGS:**

This review provides evidence for the vulnerability of foetal gonads to cancer therapy agents and the potentially damaging effects of *in utero* exposure on gonadal development and reproductive health. We hope these findings help raise awareness for the need of long-term follow-up studies to explore whether fertility is impacted in people who were exposed to cancer agents *in utero* and to identify whether they may require fertility preservation strategies.

**STUDY FUNDING/COMPETING INTEREST(S):**

No specific funding was received for this study. R.T.M. is supported by a UK Research and Innovation (UKRI) Future Leaders Fellowship (Grant Reference: MR/Y011783/1). The authors declare that they have no conflicts of interest.

**REGISTRATION NUMBER:**

Study protocol—PROSPERO (RD42021272882 and CRD42021271892).

WHAT DOES THIS MEAN FOR PATIENTS? The number of women being diagnosed with cancer during pregnancy has risen in recent decades, which also coincides with an increase in the use of chemotherapy-based treatments. However, cancer treatments are not specific to targeting only cancer cells and can cause damage to otherwise healthy body cells, including those in the male and female reproductive organs, often causing fertility problems in young cancer patients. Whilst cancer treatments are considered safe after the first trimester of pregnancy, less is known about long-term impacts on the baby, including those on reproductive health. Improved understanding of whether and how cancer therapies might impact the long-term development and reproductive health of an unborn child is key to informing both the patients and the doctors so that counselling can be offered and autonomous decisions made about treatment. Appropriate preventative measures, such as fertility preservation, could also be offered to those who have already been exposed to cancer treatments in the womb. This systematic review searched for all studies reporting on the impact of cancer therapy exposure on the reproductive health of the foetus, in studies using both animal models of exposure as well as those using human cells or tissues. When taken together, the results from these studies suggest that the developing reproductive organs could be vulnerable to cancer therapy exposure, with different types of drug exposures affecting reproductive organs and their various cell types differently, but with a consistent damaging effect. This suggests that exposure to cancer therapy in the womb could damage reproductive organs in the foetus, possibly impacting future long-term reproductive health and fertility. Therefore, long-term follow-up studies on children who have been exposed to cancer therapies in the womb are urgently required to better understand the impact and to provide appropriate treatment options or counselling.

## Introduction

Cancer is diagnosed in 1–2 out of every 1000 pregnancies, estimated to lead to the treatment of around 5000 pregnant cancer patients in Europe every year (Van Calsteren and Amant, 2014; de Haan *et al.*, 2018). Since oncological and obstetrical data are not routinely linked, the actual incidence may be underestimated (Maggen *et al.*, 2020), but the age of first-time parents in Western societies has been steadily increasing since the 1950s, so it is predicted that the rate of cancer diagnoses in pregnancy will continue to rise accordingly.

The most common malignancies diagnosed during pregnancy are breast, cervical, and lymphoma (de Haan *et al.*, 2018), with breast cancer accounting for 40% of cancers diagnosed during pregnancy (de Haan *et al.*, 2018; Maggen *et al.*, 2020). Breast cancer in pregnant women is often diagnosed at a more advanced stage, possibly due to the pregnancy-associated changes in breast weight, firmness, and density, all of which may impact the initial interpretation of the tumour as well as complicate the clinical examinations and mammogram results (Durrani *et al.*, 2018). The type of cancer treatment administered to a pregnant cancer patient depends on the type and stage of cancer but can involve chemotherapy, hormone therapy, immunotherapy, radiotherapy, or surgery, or a combination of these (Cardonick and Iacobucci, 2004). For those who are pregnant when receiving a cancer diagnosis, though, treatment can often not wait until after birth (Maggen *et al.*, 2019). The decision whether or not to treat a pregnant cancer patient requires careful consideration, where the benefit of cancer treatment must be weighed against the potential risk to the foetus, bearing in mind that studies have shown that many chemotherapy drugs have the ability to pass through the placenta and enter the foetal circulation (Van Calsteren *et al.*, 2011; Dekrem *et al.*, 2013; Köhler *et al.*, 2015; Benoit *et al.*, 2021).

Approximately 28% of all cancer patients (including non-pregnant patients) end up receiving chemotherapy treatment (Cancer Research UK, 2014), although this will vary depending on the cancer type, treatment, and patient’s age. For example, in people diagnosed with breast cancer, the proportion who subsequently receive chemotherapy treatment is estimated to be considerably higher, around 70% (Morimoto *et al.*, 2010). Cancer treatment regimens usually involve the administration of different chemotherapy agents in combination, each drug acting through contrasting mechanisms (Blommaert *et al.*, 2020). Amalgamating different chemotherapy drugs during a treatment regimen lowers the chances of the development of drug-resistant cancer cells while also providing different anti-cancer benefits to reduce tumour size and prevent metastatic potential (Mokhtari *et al.*, 2017). Chemotherapy agents primarily act by targeting proliferating malignant cancer cells in the body, but the non-specific nature of chemotherapy agents and poor selectivity for cancerous tissues mean that treatment will often result in a wide range of side effects, including detrimental effects on fertility.

Hormonal therapy treatments are also used to treat hormone-sensitive cancers, such as oestrogen receptor (ER)-positive breast cancer (NHS, 2024). Although an international consensus panel in 2010 advised against the use of hormonal therapies for cancer treatment during pregnancy (Amant *et al.*, 2010; Silverstein *et al.*, 2020), there have been numerous cases of pregnant women receiving hormonal-based anti-cancer therapies prior to this or inadvertently falling pregnant during hormonal treatment (Barthelmes and Gateley, 2004; Buonomo *et al.*, 2020).

Until relatively recently, chemotherapy was not frequently used in the treatment of pregnant cancer patients due to concerns of adverse effects to the foetus (Doll *et al.*, 1990; Cardonick and Iacobucci, 2004). Indeed, administration of chemotherapy agents is contraindicated in the first trimester of pregnancy due to increased risk of birth malformations and miscarriage (Cardonick and Iacobucci, 2004). However, delaying treatment can also worsen the prognosis. Reassuringly, when treatment is administered during the second and third trimesters of pregnancy, clinical cohort studies investigating the effects of *in utero* chemotherapy exposure have not found any association with severe congenital, neurological, or cardiac outcomes in the children born (Amant *et al.*, 2012; Esposito *et al.*, 2016; de Haan *et al.*, 2018; Blommaert *et al.*, 2020; Wolters *et al.*, 2021), nor is there evidence of increased incidences of adverse pregnancy outcomes, such as late miscarriage, for those undergoing treatment (Maggen *et al.*, 2020). However, there is a higher incidence of complications such as preterm birth and foetal growth restriction reported (Maggen *et al.*, 2021), both of which are associated with delayed neurological development and comorbidities later in life (Blommaert *et al.*, 2020). Overall, most anticancer drugs are now thought to be safe to administer after the first trimester of pregnancy, which has led to an increase in the number of cancer patients who have received cancer treatment whilst pregnant (de Haan *et al.*, 2018). Despite this research, there is a distinct lack of longer-term studies that have followed up on the potential detrimental impacts of *in utero* cancer treatments on additional health outcomes of children, in part due to the difficulty of studying these effects within the human foetus and children (Murthy *et al.*, 2014; Korakiti *et al.*, 2020; Greiber *et al.*, 2022). Even in studies that do investigate longer-term outcomes, little, if any, information is provided on the potential reproductive impact of *in utero* chemotherapy exposure. There may be many reasons for the paucity of long-term follow-up, including a lack of funding or of awareness of potential fertility implications: *in utero*-exposed individuals may not wish to participate in such studies, or perhaps the majority of the individuals are too young for their reproductive health to be definitively assessed (Murthy *et al.*, 2014). Regardless, the lack of data on this matter is concerning given the well-documented damaging effects of chemotherapy treatment on gonadal function in children and adults (Anderson *et al.*, 2015; Picton *et al.*, 2015; Allen *et al.*, 2018; Jayasinghe *et al.*, 2018; Van Dorp *et al.*, 2018; Spears *et al.*, 2019), and with long-term follow-up studies showing a significant reduction in the number of offspring born to cancer survivors (Chow *et al.*, 2016).

**Figure 1.**
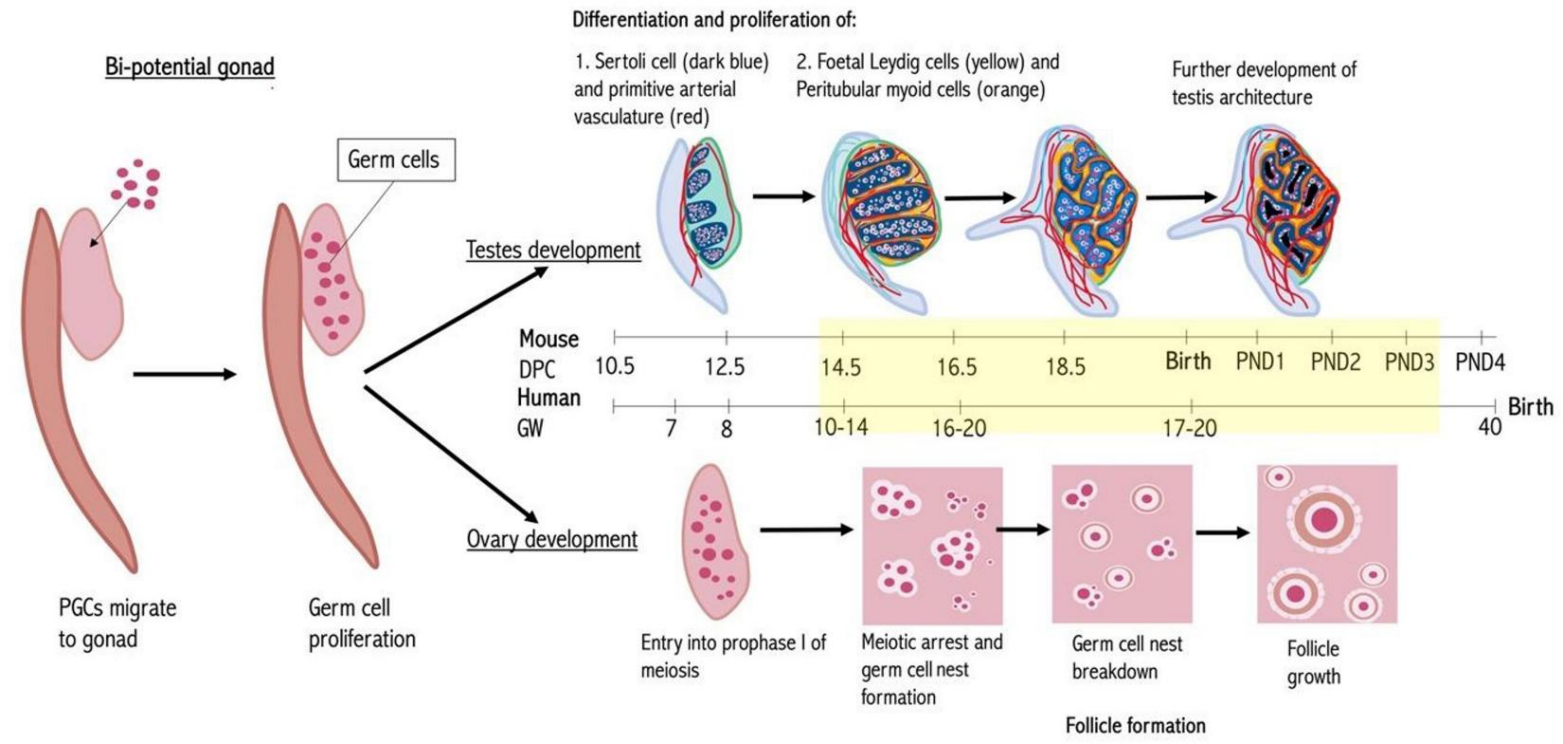
**Developmental stages of ovarian and testicular development in the mouse and human.** Yellow shaded area: the time period considered safe to administer chemotherapy during pregnancy (during the second trimester). PGCs, primordial germ cells; DPC, days post-coitum; GW, gestational week; PND, postnatal day. Adapted from figures and information in Svingen and Koopman (2013), Hartshorne *et al.* (2009), Allen *et al.* (2018), and Ozcan *et al.* (2023).

The pre-natal period of mammalian gonadal development is sensitive, complex, and crucial (Fig. 1). It initiates during foetal life with the formation of primordial germ cells (PGCs), which invade the developing gonadal ridge and proliferate rapidly. In the ovary, PGCs initiate meiosis, then form primordial follicles (PMFs) (Hartshorne *et al.*, 2009). PMFs become abundant in the human ovary from around 16–20 weeks of gestation (embryonic Day 16.5 [E16.5] in the mouse), and all PMFs will have formed before the end of the third trimester. The number of PMFs present at birth represents the pool from which all future ovulated follicles will come, i.e. the ovarian reserve. The PMF pool is therefore directly related to the future fertility and reproductive lifespan of that individual. In the developing foetal testis, Sertoli cells appear from 7 weeks of gestation (E10.5 in the mouse) and act as the central drivers of testis differentiation (Svingen and Koopman, 2013). They differentiate and engulf the PGCs to form seminiferous cords (Allen *et al.*, 2018). The germ cells (gonocytes) then differentiate into pre-spermatogonia, while the Sertoli cells, the somatic cells of the testis that are essential for supporting spermatogenesis in later life, actively proliferate during foetal life (Allen *et al.*, 2018). Their rate of proliferation at this stage and again at puberty will influence the size of the adult population of Sertoli cells and the quantity of sperm produced. The Sertoli cells also drive the differentiation of the foetal Leydig cells, which produce testosterone, and later on, also drive the development of peritubular myoid cells. Testosterone production is key for the masculinization process of the foetal testes and, in particular, during the critical period of masculinization programming window (MPW) around 8–14 weeks gestation. Impaired testosterone production or insult to key cells during this sensitive period can have a detrimental impact on male reproductive health (Sharpe, 2020).

Any compound with the ability to interfere with these vulnerable early stages of gonadal development, such as chemotherapeutic or hormonal therapies, has the potential to induce detrimental effects on the subsequent fertility of an *in utero*-exposed individual. Given the well-known gonadotoxicity of many chemotherapy drugs (Allen *et al.*, 2018; Spears *et al.*, 2019), there is a concerning possibility that children born to mothers who had chemotherapy treatment during pregnancy might have fertility complications later in life. However, only a handful of studies to date have explored the effect of chemotherapy agents on the foetal gonads. Therefore, this review aimed to systematically search for all studies reporting on the reproductive outcomes following chemotherapy or hormone therapy exposure to developing gonads. The work reviews the current evidence base for the possible impact of anti-cancer drugs on the developing gonads and/or on reproductive outcomes in both human and animal model studies to understand the impact of *in utero* chemotherapy or hormone therapy exposure on foetal reproductive development.

## Methods

The protocol for this systematic review was registered *a priori* with the PROSPERO register (CRD42021272882 and CRD42021271892) and followed the PRISMA 2020 guidelines for systematic reviews (Page *et al.*, 2021). Since this review sought to evaluate evidence from both animal model and human-based studies, two separate text searches were conducted, each with appropriate inclusion and exclusion criteria. The full search strategy is presented in Supplementary File S1.

### Inclusion and exclusion criteria

Both *in vivo* and *in vitro* study designs for human and animal model-based research were included. For inclusion in the review, a study had to contain outcomes relating to the reproductive health or fertility of the exposed groups; studies were excluded if an appropriate control was not used. Human-based studies were included if foetal tissue or individuals were exposed to any chemotherapy drug *in utero* or during the prenatal period (and had not been exposed to any other cancer treatment such as radiotherapy). Studies were excluded if there had been any exposure of the child to chemotherapy after birth. For *in vitro* experiments investigating pro-drugs, papers were only included where the active metabolite(s) were added to the medium. Animal-model studies in rodents were included if the exposure (*in vivo* or *in vitro*) was up to and including postnatal Day 4 (PND4), which has been shown in rodents to be the equivalent to human foetal gonadal development towards the end of the third trimester (Hartshorne *et al.*, 2009; Stefansdottir *et al.*, 2016; Allen *et al.*, 2018). All included studies were conducted on mammalian tissues, specifically human, mouse, or rat.

### Search strategy

A keyword search was used to return animal and human studies from the databases PubMed, Web of Science, and Google Scholar, with publication dates up to July 2024 (Supplementary File S1). The returned articles from each database were then imported into Covidence reference manager software (Covidence, 2024), where duplicates were removed, and the final number for title and abstract screening was presented. Screening, using the inclusion and exclusion criteria, was carried out separately for the animal model and human studies. Two independent reviewers undertook the first title and abstract and then full-text screening stages. Any discrepancies were discussed between reviewers to decide on final inclusion or exclusion. The final included studies from both the animal model and human searches underwent independent data extraction by two reviewers and were then merged into a single spreadsheet collated from the agreed data.

### Data extraction and synthesis

The data extracted for both animal model and human studies included study characteristics: year of publication, study design, methods, aim, primary outcomes, and key findings. The specific drug exposure and reproductive outcomes of interest were then extracted. This included sample or population information, intervention and chemotherapy used, timing of administration, dosage, and the findings of reproductive outcomes measured in the study.

Data on *in utero* hormonal therapy use and reproductive outcomes were also extracted, as this was used as treatment for pregnant women in some cases before it was advised against in 2010 (Silverstein *et al.*, 2020). The key outcomes of each article were summarized in a main table of reproductive outcomes and tabulated by chemotherapy or hormonal drug and class. Heterogeneity within the reporting of data and limited comparative values meant that meta-analyses of the data were not possible. Given that, narrative synthesis of the trends seen and discussion of future recommendations in light of the results presented was undertaken. Results from both human and animal model studies were grouped together for interpretation. Direction of effect (significant differences) and change were presented for each outcome extracted and merged for those studies reporting the same effect. The studies investigating hormonal therapies and their outcomes were collated in a separate table. Data from each study were grouped by drug and analysed narratively to examine the evidence presented for adverse reproductive outcomes following cancer treatment exposure *in utero.*

### Risk of bias and quality assessment

All included studies were assessed by the independent researchers for possible sources of bias and for quality of results. Comments on the validity and justification of conclusions, as well as the methods used, were recorded in the characteristics table. Each included study was also assessed for quality and bias using the validated tool, SciRAP tool for *in vivo* toxicology studies—assessment sheet or SciRAP tool for *in vitro* toxicology studies—assessment sheet, where appropriate, versions 2.3 (Karolinska Institute, 2024). The final score for each study was included in the study characteristics table (Supplementary Table S1). One study was removed following full-text inclusion due to poor-quality methods and results (Saxena and Singh, 1999). The SciRAP tool does not include a specific tool to categorize the reliability of the studies from their scores; therefore, we established a method of converting these scores into reliability groups, as found in other studies using this tool (Wiklund and Beronius, 2022).

## Results

**Figure 2.**
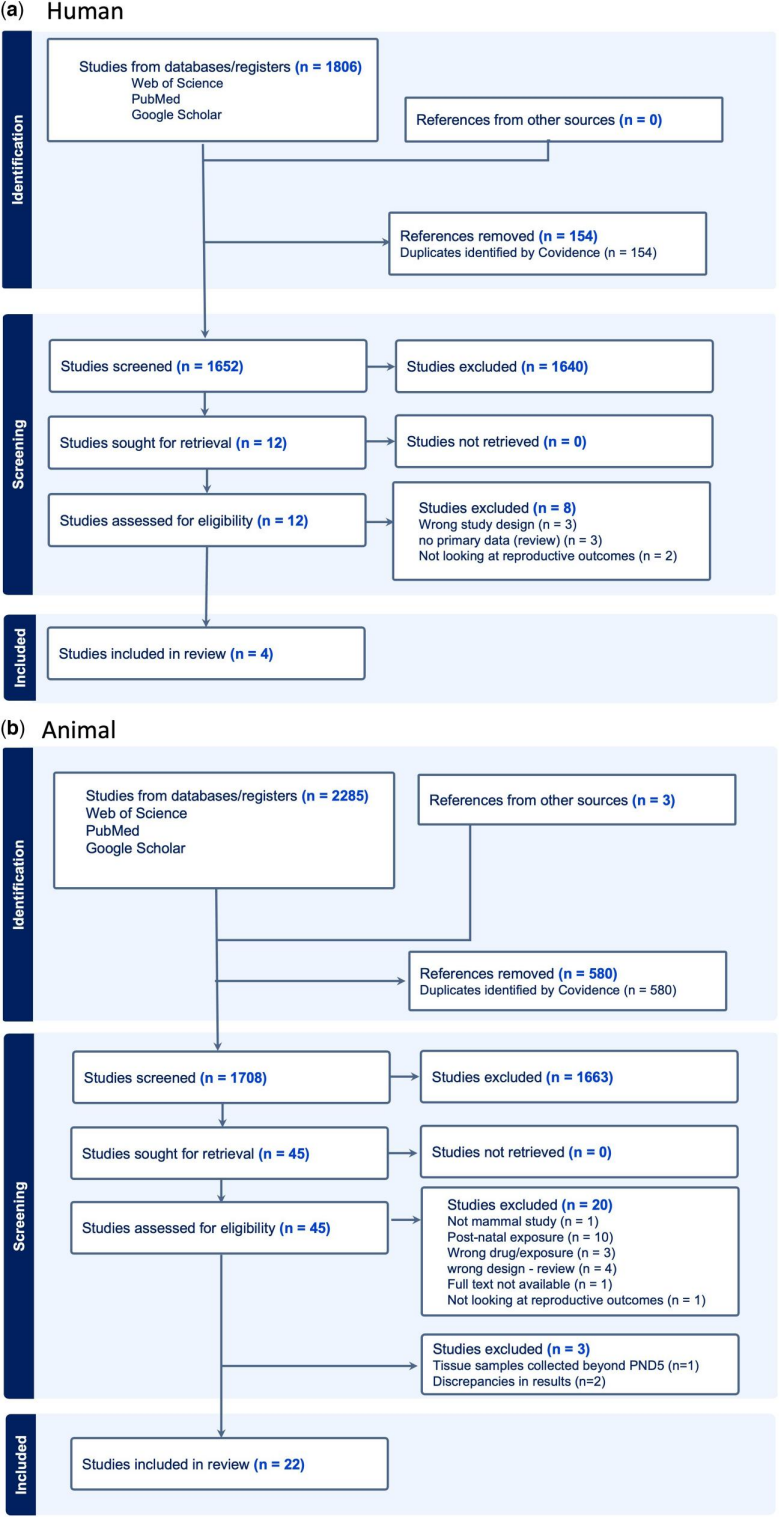
**PRISMA diagram.** Results of search strategy and screening in this systematic review, detailing both (**a**) human and (**b**) animal studies. Both searches looked for studies of chemotherapy and hormonal therapy exposures. Created using Covidence reference software (Covidence, 2024).

The stages of study selection and screening are presented in the PRISMA diagrams (Fig. 2). Following full-text review, one study that initially met our study criteria was excluded (Yu *et al.*, 2014), as it demonstrated fundamental discrepancies between described tissues and the figures included, which discredited their results to the reviewers.

Four human-based studies met the inclusion criteria, along with 22 animal model-based studies (total n = 26). The full data extraction table with characteristics of included studies and outcomes of interest can be found in Supplementary Table S1. Based on their SciRAP methodological scores (Supplementary Table S2), 24 of the included studies were classed as reliable, or reliable with restrictions. Two of the included studies were classed as having low reliability.

### Treatment with chemotherapy and hormone therapy

The studies included in this review investigated exposure to a range of clinically relevant chemotherapy agents and hormone-based therapies that may be used for the treatment of pregnant cancer patients, outlined in Table 1.

**Table 1.** The cancer drugs used in included studies and the evidence for their placental transfer rate.

Cancer drug (drug class)	Studies (n)		Placental transfer	
	Human (n = 4)	Animal model (n = 22)	Human data (*in vivo* and *ex vivo*)	Mammalian data
**Chemotherapy**				
Cisplatin (platinum, alkylating agent)	4	1	FTR = 9% (*ex vivo*). Platinum concentrations in umbilical cord blood and amniotic fluid were 23–65% and 11–42%, respectively, when compared to maternal blood.	Pata monkeys/rat models: detected in foetal and neonate’s tissues.
Carboplatin (platinum, alkylating agent)	1		FTR = 4–13% (*ex vivo*)	Mouse model: FTR = 117%, baboon model: FTR = 57.5%
Cyclophosphamide (alkylating agent)		4	Detected in amniotic fluid: 25% of maternal plasma level 1 h after injection (*in vivo*).	Baboon model: FTR = 25%
Etoposide (podophyllotoxin derivative)		1	No data	Mouse model: induced apoptosis in trophoblasts. No data on FTR
Cytarabine (antimetabolite)		1	No data on FTR	No data on FTR, transplacental transport documented in mice
Busulfan (alkylating agent)		1	No data	No data
Doxorubicin		1	FTR of pegylated liposomal doxorubicin = 0%, FTR of non-pegylated liposomal doxorubicin = 12%. Detectable in foetal organs at the delivery in human *in-vivo* studies, but not in cord blood, amniotic fluid or placenta.	Average materno-foetal transfer rate ranged between 4% and 7.5%.
Paclitaxel		1	FTR = 1.72–7% (*ex vivo* data).	Mouse model: no evidence of placental transfer, baboon model: FTR = 1.5% detectable in foetal tissues.
Docetaxel		2	FTR = 4% (*ex vivo*)	Baboon model: undetectable in foetal blood, detected on foetal tissues.
**Hormone therapy**				
Tamoxifen (hormone receptor antagonist)		12	No data	No data
Letrozole (aromatase inhibitor hormone antagonist)		1	No data	No data

Some studies may have used more than one drug in their exposures. Total number of studies = 26, evidence from placental transfer data taken from Köhler *et al.* (2015), Benoit *et al.* (2021), and Triarico *et al.* (2022). FTR, foetal transfer rate.

### Reproductive effects of *in utero* chemotherapy drug exposure

A total of 13 studies examined the impact of chemotherapy agents on foetal gonadal and/or reproductive health, together reporting on 23 different reproductive outcomes that included analyses of gonadal cell type number and health, hormone levels, anogenital distance (a readout of foetal testosterone production), and levels of gonadal apoptosis and oxidative stress (summarized in Table 2).

**Table 2.** Reproductive outcomes and direction of effect following *in utero* exposure to chemotherapy drugs.

Outcome	Direction of effect	Treatment drug	Stage of exposure/duration	Dose tested	Timing of analysis post-exposure	Tissue and/or cell type	Experimental design	Species (strain, where relevant)	Reference
**Germ cell number**	**↓**	Carboplatin	24 h, second trimester	5 µg/ml	240 h	Testis	*In vitro*	Human	Tharmalingam *et al.*, 2020
	**↓**	Cisplatin	24 h, second trimester (14–22 weeks)	0.5 and 1.0 μg/ml	72 and 96 h	Testis	*In vitro*	Human	Tharmalingam *et al.*, 2020
	**↓**	Cisplatin	24 h, second trimester (17–19 GW)	0.5 µg/ml	72 h	Testis	*In vitro*	Human	Matilionyte *et al.*, 2022a
	**↓**	Cisplatin	24 h, second trimester (17–22 GW)	0.5 µg/ml	240 h	Testis	*In vitro*	Human	Matilionyte *et al.*, 2023
	**↓**	Cisplatin	24 h, Day 1–2 of culture (PND1 equivalent)	5 µg/ml	0 or 96 h (Day 2 or 6 of culture)	Ovary	*In vitro*	Mouse (C57BL/6)	Morgan *et al.*, 2013
	**↓**	Cyclophosphamide	Single dose on days E10.5 and E11.5	7.5 mg/kg	PND7	Ovary	*In vivo*	Mouse (129/L1)	Comish *et al.*, 2014
	**↓**	Docetaxel	24 h, Day 1–2 of culture (PND1 equivalent)	0.1, 1, 10 μM	96 h (Day 6 of culture)	Ovary	*In vitro*	Mouse (C57BL/6)	Lopes *et al.*, 2014
	**↓**	Doxorubicin	24 h, Day 1–2 of culture (PND1 equivalent)	0.01, 0.05, 0.1, 0.2 μg/ml	0 or 96 h (Day 2 or 6 of culture)	Ovary	*In vitro*	Mouse (C57BL/6)	Morgan *et al.*, 2013
	**↓**	Doxorubicin	48 h, from Day 2 of culture (PND2 equivalent)	1.8, 3.6 μg/ml	0 or 72 h (Day 4 or 7 of culture)	Ovary	*In vitro*	Mice (C57BL/6)	Ozcan *et al.*, 2023
	**↓**	Etoposide	6 days from PND0 (Day 0–6 of culture)	50, 100, 150, or 200 ng/ml	0 h (Day 6 of culture)	Ovary	*In vitro*	Mouse (CD1)	Stefansdottir *et al.*, 2016
	**↓**	Etoposide	6 days from E13.5 (Day 0–6 of culture)	50–150 ng/ml	0 h and 6 days (Day 6 or 12 of culture)	Ovary	*In vitro*	Mouse (CD1)	Stefansdottir *et al.*, 2016
	**↔**	Docetaxel and paclitaxel	48 h, from Day 2 of culture (PND2 equivalent)	Doc: 2.2, 4.4 μg/ml Pac: 1.8, 3.6 μg/ml	PND4 and 7	Ovary	*In vitro*	Mice (C57BL/6)	Ozcan *et al.*, 2023
	**↔**	Paclitaxel	Single dose, E16.5	100 mg/m^2^	PND14 and 30	Ovary	*In vivo*	Mouse (C57BL/6)	Chaqour *et al.*, 2024
	**↔**	Docetaxel	Single dose, E16.5	100 mg/m^2^	PND14 and 30	Ovary	*In vivo*	Mouse (C57BL/6)	Chaqour *et al.*, 2024
	**↔**	Cisplatin	24 h, second trimester (14–16 and 20–22 GWs)	0.5 µg/ml	72 h	Testis	*In vitro*	Human	Matilionyte *et al.*, 2022a
	**↔**	Cisplatin	24 h, second trimester (14–22 weeks)	0.5 and 1.0 μg/ml	24 h	Testis	*In vitro*	Human	Tharmalingam *et al.*, 2020
	**↔**	Carboplatin	24 h, second trimester (14–22 weeks)	0.5 and 1.0 μg/ml	72 h	Testis	*In vitro*	Human	Tharmalingam *et al.*, 2020
**Sperm count**	**↓**	Cyclophosphamide	Single dose on days E10.5 and E11.5	7.5 mg/kg	8-week-old adult males	Testis	*In vivo*	Mouse (129/L1)	Comish *et al.*, 2014
**Leydig cell number**	**↓**	Cytarabine	Daily dose between GD8 and 21	12.5 or 25 mg/kg/day	PND1	Testis (reduction in size and number of supporting cells)	*In vivo*	Rat	Namoju and Chilaka, 2022
	**↔**	Busulfan	Single dose, GD13	10 mg/kg	PND21, 40 and 60	Testis	*In vivo*	Rat (Wistar)	Hall and Gomes, 1973
**Sertoli cell number**	**↔**	Cisplatin	24 h, second trimester	0.5 and 1.0 μg/ml	24, 96 h, and 12 weeks	Testis (Sertoli cell number or function)	*In vitro*	Human	Matilionyte *et al.*, 2022b
	**↔**	Busulfan	Single dose on GD13	10 mg/kg	PND21, 40 and 60	Testis	*In vivo*	Rat (Wistar)	Hall and Gomes, 1973
**Apoptosis**	**↑**	Cisplatin	24 h, second trimester	0.5 µg/ml	72 h	Testis (Sertoli cells)	*In vitro*	Human	Matilionyte *et al.*, 2022b
	**↑**	Cisplatin	24 h, Day 1–2 of culture (PND1 equivalent)	0.1, 0.5, 1, 5 µg/ml	0 or 96 h (Day 2 or 6 of culture)	Ovary	*In vitro*	Mouse (C57BL/6)	Morgan *et al.*, 2013
	**↑**	Docetaxel	24 h, Day 1–2 of culture (PND1 equivalent)	0.1, 1, 10 μM	96 h (Day 6 of culture)	Ovary	*In vitro*	Mouse (C57BL/6)	Lopes *et al.*, 2014
	**↑**	Docetaxel	Single dose, E16.5	100 mg/m^2^	PND14 and 30	Ovary	*In vivo*	Mouse (C57BL/6)	Chaqour *et al.*, 2024
	**↑**	Paclitaxel	Single dose, E16.5	175 mg/m^2^	PND14 and 30	Ovary	*In vivo*	Mouse (C57BL/6)	Chaqour *et al.*, 2024
	**↑**	Doxorubicin	24 h, Day 1–2 of culture (PND1 equivalent)	0.01, 0.05, 0.1, 0.2 μg/ml	0 or 96 h (Day 2 or 6 of culture)	Ovary	*In vitro*	Mouse (C57BL/6)	Morgan *et al.*, 2013
	**↔**	Cisplatin	24 h, second trimester (14–22 weeks)	0.5 and 1.0 μg/ml	24 and 96 h	Testis	*In vitro*	Human	Tharmalingam *et al.*, 2020
	**↔**	Cisplatin	24 h, second trimester	0.5 µg/ml	240 h	Testis (Sertoli cells)	*In vitro*	Human	Matilionyte *et al.*, 2022b
**Oxidative stress**	**↑**	Cytarabine	Daily dose between GD8 and 21	12.5 or 25 mg/kg/day	PND1	Testis	*In vivo*	Rat	Namoju and Chilaka, 2022
**Testis weight**	**↓**	Cyclophosphamide	Single dose on days E10.5 and E11.5	7.5 mg/kg	8 weeks (adult males)	Testis	*In vivo*	Mouse (129/L1)	Comish *et al.*, 2014
	**↓**	Cytarabine	Daily dose between GD8 and 21	12.5 or 25 mg/kg/day	PND1	Testis (dose dependent)	*In vivo*	Rat	Namoju and Chilaka, 2022
	**↓**	Busulfan	Single dose on GD13	10 mg/kg	PND60	Testis (+ seminal vesicle weight in PND40 and 60)	*In vivo*	Rat (Wistar)	Hall and Gomes, 1973
**Anogenital distance**	**↓**	Cytarabine	Daily dose between GD8 and 21	12.5 or 25 mg/kg/day	PND1	Testis (dose dependent)	*In vivo*	Rat	Namoju and Chilaka, 2022
**AMH concentration**	**↔**	Cisplatin	24 h, second trimester	0.5 and 1.0 µg/ml	24 and 96 h	Testis	*In vitro*	Human	Matilionyte *et al.*, 2022b
	**↔**	Paclitaxel	Single dose, E16.5	175 mg/m^2^	PND30	Ovary	*In vivo*	Mouse (C57BL/6)	Chaqour *et al.*, 2024
	**↔**	Docetaxel	Single dose, E16.5	100 mg/m^2^	PND30	Ovary	*In vivo*	Mouse (C57BL/6)	
**LH concentration**	**↔**	Paclitaxel	Single dose, E16.5	175 mg/m^2^	PND30	Ovary	*In vivo*	Mouse (C57BL/6)	Chaqour *et al.*, 2024
	**↔**	Docetaxel	Single dose, E16.5	100 mg/m^2^	PND30	Ovary	*In vivo*	Mouse (C57BL/6)	
**FSH concentration**	**↓**	Paclitaxel	Single dose, E16.5	175 mg/m^2^	PND30	Ovary	*In vivo*	Mouse (C57BL/6)	Chaqour *et al.*, 2024
	**↑**	Docetaxel	Single dose, E16.5	100 mg/m^2^	PND30	Ovary	*In vivo*	Mouse (C57BL/6)	
**Testosterone level**	**↓**	Cytarabine	Daily dose between GD8 and 21	12.5 or 25 mg/kg/day	PND1	Testis	*In vivo*	Rat	Namoju and Chilaka, 2022
	**↔**	Busulfan	Single dose GD13	10 mg/kg	PND60	Testis	*In vivo*	Rat (Wistar)	Hall and Gomes, 1973
	**↑**				PND21 and 40	Testis			
**Disrupted testicular architecture**	**↑**	Cyclophosphamide	Single dose on days E10.5 and E11.5	7.5 mg/kg/day	4 weeks	Testis	*In vivo*	Mouse (129/L1)	Comish *et al.*, 2014
	**↑**	Cytarabine	Daily dose between GD8 and 21	12.5 or 25 mg/kg/day	PND1	Testis	*In vivo*	Rat	Namoju and Chilaka, 2022
	**↑**	Busulfan	Single dose on GD13	10 mg/kg	PND21, 40 and 60	Testis	*In vivo*	Rat (Wistar)	Hall and Gomes., 1973
**Follicle activation**	**↑**	Cyclophosphamide	Single dose on days E10.5 and E11.5	7.5 mg/kg	PND7	Ovary	*In vivo*	Mouse (129/L1)	Comish *et al.*, 2014
**Meiotic disruption**	**↑**	Etoposide	6 days, from E13.5	50, 100, 150 200 ng/ml	Day 2 of culture	Ovary	*In vitro*	Mouse (CD1)	Stefansdottir *et al.*, 2016
**Multi-oocyte ** **follicles**	**↑**	Docetaxel	Single dose, E16.5	100 mg/m^2^	PND14, 30	Ovary	*In vivo*	Mouse (C57BL/6)	Chaqour *et al.*, 2024
	**↔**	Paclitaxel	Single dose, E16.5	175 mg/m^2^	PND14, 30	Ovary	*In vivo*	Mouse (C57BL/6)	
**Number of oocytes retrieved after ** **super-ovulation**	**↓**	Docetaxel	Single dose, E16.5	100 mg/m^2^	PND30	Ovary	*In vivo*	Mouse (C57BL/6)	Chaqour *et al.*, 2024
	**↓**	Paclitaxel	Single dose, E16.5	175 mg/m^2^	PND30	Ovary	*In vivo*	Mouse (C57BL/6)	
**Number of litters**	**↓**	Docetaxel	Single dose, E16.5	100 mg/m^2^	PND30 + 1 year	Ovary	*In vivo*	Mouse (C57BL/6)	Chaqour *et al.*, 2024
	**↓**	Paclitaxel	Single dose, E16.5	175 mg/m^2^	PND30 + 1 year	Ovary	*In vivo*	Mouse (C57BL/6)	
**Total pups per ** **litter**	**↔**	Docetaxel	Single dose, E16.5	100 mg/m^2^	PND30 + 1 year	Ovary	*In vivo*	Mouse (C57BL/6)	Chaqour *et al.*, 2024
	**↔**	Paclitaxel	Single dose, E16.5	175 mg/m^2^	PND30 + 1 year	Ovary	*In vivo*	Mouse (C57BL/6)	
**Live pups per litter**	**↔**	Docetaxel	Single dose, E16.5	100 mg/m^2^	PND30 + 1 year	Ovary	*In vivo*	Mouse (C57BL/6)	Chaqour *et al.*, 2024
	**↔**	Paclitaxel	Single dose, E16.5	175 mg/m^2^	PND30 + 1 year	Ovary	*In vivo*	Mouse (C57BL/6)	
**Age at final litter**	**↔**	Docetaxel	Single dose, E16.5	100 mg/m^2^	PND30 + 1 year	Ovary	*In vivo*	Mouse (C57BL/6)	Chaqour *et al.*, 2024
	**↓**	Paclitaxel	Single dose, E16.5	175 mg/m^2^	PND30 + 1 year	Ovary	*In vivo*	Mouse (C57BL/6)	
**Folliculogenesis in F2 generation**	**↔**	Docetaxel	Single dose, E16.5	100 mg/m^2^	PND14 in F2 generation	Ovary	*In vivo*	Mouse (C57BL/6)	Chaqour *et al.*, 2024
	**↔**	Paclitaxel	Single dose, E16.5	175 mg/m^2^	PND14 in F2 generation	Ovary	*In vivo*	Mouse (C57BL/6)	
**Follicle atresia in F2 generation**	**↑**	Docetaxel	Single dose, E16.5	100 mg/m^2^	PND14 in F2 generation	Ovary	*In vivo*	Mouse (C57BL/6)	Chaqour *et al.*, 2024
	**↔**	Paclitaxel	Single dose, E16.5	175 mg/m^2^	PND14 in F2 generation	Ovary	*In vivo*	Mouse (C57BL/6)	

Up/down arrows denote significant differences in outcome to control groups (*P*<0.05). The horizontal bidirectional arrow denotes no significant difference. E, embryonic day; F2, second filial generation; GW, gestational week; PND, postnatal day; GD, gestational day.

Seven of those studies examined foetal testicular effects, and six of the studies examined effects on the foetal ovary. Four of these studies were based on human tissues, whilst the other nine used rodent models (rats or mice). Of these, several studies demonstrated a significant decrease in germ cell number following chemotherapy exposure, in both human and rodent studies (Morgan *et al.*, 2013; Comish *et al.*, 2014; Lopes *et al.*, 2014; Stefansdottir *et al.*, 2016; Tharmalingam *et al.*, 2020; Matilionyte *et al.*, 2022a, 2023). This significant decrease was reported in the human foetal testis following *in vitro* exposure to both cisplatin and carboplatin (Tharmalingam *et al.*, 2020; Matilionyte *et al.*, 2022a, 2023), as well as the mouse ovary after *in vivo* exposure to cyclophosphamide (Comish *et al.*, 2014) and *in vitro* exposure to etoposide, doxorubicin, docetaxel, and cisplatin (Morgan *et al.*, 2013; Lopes *et al.*, 2014; Stefansdottir *et al.*, 2016; Ozcan *et al.*, 2023). One study found a detrimental impact on testicular Leydig cell number and consequently, reduced testosterone levels, following cytarabine exposure (Namoju and Chilaka, 2022). The Sertoli cell population was unchanged in cultured second-trimester human testis tissue treated with cisplatin (Matilionyte *et al.*, 2022b), or after *in vivo* busulfan exposure to the foetal rat testis (Hall and Gomes, 1973). Increased levels of apoptosis were observed in second-trimester human foetal testicular gonadal tissue following cisplatin exposure (Matilionyte *et al.* 2022b). Mouse and rat testis weights were also significantly reduced, and testicular architecture was disrupted by the chemotherapy agents cyclophosphamide, cytarabine, and busulfan *in vivo* (Hall and Gomes, 1973; Comish *et al.*, 2014; Namoju and Chilaka, 2022).

In the ovary, *in utero* cyclophosphamide increased follicular activation once mice had reached adulthood (Comish *et al.*, 2014). In another *in vitro* exposure study of the foetal mouse ovary, etoposide caused meiotic disruption and germ cell loss, which subsequently impacted the number of follicles formed (Stefansdottir *et al.*, 2016). Furthermore, doxorubicin exposure at PND2 caused a 91% and 95% loss of oocyte density compared to controls at PND4 and PND7, respectively (Ozcan *et al.*, 2023). On the other hand, this study found that exposure of mouse PND2 ovaries to cisplatin, docetaxel, and paclitaxel did not lead to significant oocyte loss compared to controls. Overall, the combined results from the included studies provide evidence that *in utero* exposure to different types of chemotherapy drugs can have a detrimental impact on many key gonadal cell populations and reproductive processes in both males and females (Table 2). However, there were also instances where chemotherapy exposure did not cause significant aberrations when compared to controls. Docetaxel and paclitaxel were not found to induce significant germ cell loss in the mouse ovary at PND14 and 30 following *in vivo* exposure on embryonic Day 16.5, although an increase in the number of atretic and apoptotic follicles was found (Chaqour *et al.*, 2024). A single exposure to busulphan at gestation Day 13 did not significantly change the number of Leydig cells or Sertoli cells in the rat testis (Hall and Gomes, 1973). Sertoli cell number was also not affected following *in vitro* cisplatin exposure of the human foetal testis (Matilionyte *et al.*, 2022b). Encouragingly, there were several instances of consistent findings between human and rodent model studies, supporting the applicability of animal studies to model human studies in this case.

### *In utero* effects of hormone therapy exposure (tamoxifen and letrozole)

Hormonal cancer therapies are used in the treatment of particular cancers, for example, hormone receptor-positive breast cancers, which have either oestrogen or progesterone receptors. For these cancers, drugs such as tamoxifen are used to inhibit the ER activity (Kazimir *et al.*, 2023). Consequently, this review also examined studies that investigated the effects of *in utero* exposure to hormonal cancer therapies (Table 3).

**Table 3.** Reproductive outcomes and direction of effect following *in utero* exposure to hormonal cancer therapies.

Outcome	Direction of effect	Treatment drug	Stage of exposure/duration	Dose received	Timing of analysis (post-exposure)	Tissue and/or cell type examined	Study design	Species	Reference
**Germ cell number**	**↓**	Tamoxifen	Single dose, 1 h after birth	200 µg	PND90	Testes (spermatogonia)	*In vivo*	Rat (Wistar)	Gonzalez-Gonzalez *et al.*, 2017
	**↓**		Single dose, GD13	100 µg	PND6 and 7 litters	Ovaries (oocyte diameter)	*In vivo*	Mouse (BALB/c)	Roshangar *et al.*, 2010
	**↓**		Single dose, E16.5	0.08, 0.8 and 8 µg/kg	3dpp	Ovaries (oocytes)—primordial follicle counts	*In vivo*	Mouse (ICR)	Zhao *et al.*, 2023
	**↓**	Letrozole	3 days, GD16-18	1.00 and 1.25 mg/kg	PND60	Testes (spermatogenesis arrest)	*In vivo*	Rat (Sprague- Dawley)	Shaaban *et al.*, 2023
	**↓**	Tamoxifen	5 days PND1–5	0.4 mg/kg	PND70	Ovary (adult)—primordial, primary, secondary and antral follicles	*In vivo*	Mouse (BALB/c)	Parandin *et al.*, 2016
**Spermatogenesis**	**↓**	Letrozole	3 days, GD16–18	0.25, 0.75, 1.00, and 1.25 mg/kg/BW	PND60	Testes	*In vivo*	Rat (Sprague- Dawley)	Shaaban *et al.*, 2023
**Corpora Lutea**	**↓**	Tamoxifen	4 days, PND0–4	25 and 50 µg/pup	1.5, 3, 6, 12, 18 months post birth	Ovaries (adult)	*In vivo*	Mouse (CD1)	Razvi *et al.*, 2007
	**↓**		5 days PND1–5	0.4 mg/kg	PND70	Ovary (adult)	*In vivo*	Mouse (BALB/c)	Parandin *et al.*, 2016
**Sertoli cell number**	**↓**	Tamoxifen	Single dose, 1 hr after birth	200 µg	PND90	Testes	*In vivo*	Rat (Wistar)	Gonzalez-Gonzalez *et al.*, 2017
**Leydig cells**	**↔**	Tamoxifen	Single dose, 1 h after birth	200 µg	PND90	Testes	*In vivo*	Rat (Wistar)	Gonzalez-Gonzalez *et al.*, 2017
**Apoptosis**	**↑**	Tamoxifen	Single dose, E16.5	0.8 and 8 µg/kg	3dpp	Ovaries (TUNEL staining, % positive cells)	*In vivo*	Mouse (ICR)	Zhao *et al.*, 2023
**Cell proliferation**	**↓**	Tamoxifen	Single dose, E16.5	0.8 and 8 µg/kg	3dpp	Ovaries (expression of edU, PCNA, and Ki67)	*In vivo*	Mouse (ICR)	Zhao *et al.*, 2023
**Testis weight**	**↓**	Tamoxifen	Single dose, 1 hr after birth	200 µg	PND90	Testes	*In vivo*	Rat (Wistar)	Gonzalez-Gonzalez *et al.*, 2017
	**↓**		5 days, PN1-5	12.5 and 100 µg	PND243	Adult testes	*In vivo*	Rat (Wistar)	Morales-Otal *et al.*, 2005
	**↓**		5 days, PN1–5	2 µg in 5 µl vehicle	PND89-95	Adult testes	*In vivo*	Mouse (A/J, AKRJJ, BALB/cAnN, C3H/HeJ, C57BL/6J, DBA/2J, and FVB)	Nakai *et al.*, 1999
	**↓**		5 days; PND1-5	14.0 mg/kg	15 months (PND456)	Adult testes	*In vivo*	Rat (Sprague- Dawley)	Karlsson, 2006
	**↑**	Letrozole	3 days, GD16–18	0.75, 1.00 and 1.25 mg/kg	PND60	Testes	*In vivo*	Rat (Sprague- Dawley)	Shaaban *et al.*, 2023
**Uterine weight**	**↓**	Tamoxifen	4 days, PND0-4	1 mg/kg	1.5, 3 and 6 months post birth	Uteri	*In vivo*	Mouse (CD1)	Green *et al.*, 2005
	**↓**		4 days, PND0-4	5, 10, 25 and 50 µg/pup	1.5, 3, 6, 12, 18 months post birth	Uteri	*In vivo*	Mouse (CD1)	Razvi *et al.*, 2007
	**↓**		Days 15–20 of gestation	20µg	PND21	Uteri	*In vivo*	Rat (Sprague- Dawley)	Hilakivi-Clarke *et al.*, 2000
	**↑**	Tamoxifen	4 days, PND0-4	1 mg/kg	12 months post birth	Uteri	*In vivo*	Mouse (CD1)	Green *et al.*, 2005
**DNA double-strand breaks**	**↑**	Tamoxifen	Single dose, E16.5	0.08, 0.8, and 8 µg/kg	21dpp	Ovaries	*In vivo*	Mouse (ICR)	Zhao *et al.*, 2023
**Disrupted uterine histology**	**↑**	Tamoxifen	5 days; PND1-5	14.0 mg/kg	15 months (PND456)	Adult uterus—luminal dilation, absence of glands, irregular myometrium	*In vivo*	Rat (Sprague- Dawley)	Karlsson, 2006
**Testosterone level**	**↑**	Letrozole	3 days, GD16–18	1.00 and 1.25 mg/kg	PND60	Testes	*In vivo*	Rat (Sprague- Dawley)	Shaaban *et al.*, 2023
	**↔**	Tamoxifen	5 days, PND1–5	12.5 and 100 µg	PND243	Adult testes	*In vivo*	Rat (Wistar)	Morales-Otal *et al.*, 2005
**Disrupted testicular architecture**	**↑**	Tamoxifen	Single dose, 1 hr after birth	200 µg	PND90	Testes	*In vivo*	Rat (Wistar)	Gonzalez-Gonzalez *et al.*, 2017
	**↑**	Letrozole	3 days, GD16-18	1.00 and 1.25 mg/kg	PND60	Testes (necrosis and disruption of epithelium of seminiferous tubules).	*In vivo*	Rat (Sprague- Dawley)	Shaaban *et al.*, 2023
	**↑**	Tamoxifen	5 days, PND1–5	2 µg in 5 µl vehicle	PND89-95	Adult testes (testicular structures and epipidymes)	*In vivo*	Mouse (C3H/HeJ, C57BL/6J, DBA/2J, A/J strains)	Nakai *et al.*, 1999
	**↑**	Tamoxifen	5 days; PND1–5	14.0 mg/kg	15 months (PND456)	Adult testes	*In vivo*	Rat (Sprague- Dawley)	Karlsson, 2006
	**↔**	Tamoxifen	5 days, PN1–5	2 µg in 5 µl vehicle	PND89–95	Adult testes	*In vivo*	Mouse (AKRJJ, BALB/cAnN, FVB/N strains)	Nakai *et al.*, 1999
**Anogenital distance**	**↑**	Letrozole	3 days, GD16–18	1.00 and 1.25 mg/kg	PND21	Testes	*In vivo*	Rat (Sprague- Dawley)	Shaaban *et al.*, 2023
**Germ cell nests**	**↑**	Tamoxifen	Single dose, GD13	100 µg	PND2 and 3	Ovaries (number of follicular nests vs separate oocytes)	*In vivo*	Mouse (BALB/c)	Roshangar *et al.*, 2010
	**↓**	Tamoxifen	GD8-13	0.4 mg/kg/day	PND5	Neonatal ovaries (formation of nests)	*In vivo*	Rat (Wistar)	Malekinejad *et al.*, 2011
**Epigenetic modifications**	**↑**	Tamoxifen	Single dose, E16.5	0.08, 0.8, and 8 µg/kg	21dpp	Ovaries	*In vivo*	Mouse (ICR)	Zhao *et al.*, 2023
**Ovary weight**	**↓**	Tamoxifen	5 days; E15–E20	20 µg	PND21	Newborn ovary	*In vivo*	Rat (Sprague- Dawley)	Hilakivi-Clarke *et al.*, 2000
			5 days; PND1–5	14.0 mg/kg	15 months (PND456)	Adult ovaries	*In vivo*	Rat (Sprague- Dawley)	Karlsson, 2006
			5 days PND1–5	0.4 mg/kg	PND70	Adult (ovary collection)	*In vivo*	Mouse (BALB/c)	Parandin *et al.*, 2016
**Ovarian follicles**	**↔**	Tamoxifen	1 day, E16.5	0.08, 0.8 µg/kg	21dpp	Ovaries (total number of follicles)	*In vivo*	Mouse (ICR)	Zhao *et al.*, 2023
	**↓**			8 µg/kg/d	21dpp	Ovaries (total number of follicles and percentage of antral follicles)			
	**↑**			8 µg/kg/d	21dpp	Ovaries (percentage of primordial follicles)			
	**↓**	Tamoxifen	Single dose, GD13	100 µg	PND6 and 7	Ovaries (number and diameter of the follicles, higher ratio of primordial vs growing follicles in experimental group).	*In vivo*	Mouse (BALB/c)	Roshangar *et al.*, 2010
	**↓**	Tamoxifen	GD8–13	0.4 mg/kg/day	E20	Foetal ovaries (differentiation of gonocytes for follicle formation)	*In vivo*	Rat (Wistar)	Malekinejad *et al.*, 2011
**Days to puberty onset**	**↓**	Tamoxifen	5 days PND1–5	0.4 mg/kg	From PND24	Vaginal opening (puberty onset)	*In vivo*	Mouse (BALB/c)	Parandin *et al.*, 2016
			Days 15–20 of gestation	20µg	PND21	Vaginal opening	*In vivo*	Rat (Sprague- Dawley)	Hilakivi-Clarke *et al.*, 2000
**Number of oestrous cycles**	**↓**	Tamoxifen	5 days PND1–5	0.4 mg/kg	From PND24 - 70	Vaginal lavage (oestrus)	*In vivo*	Mouse (BALB/c)	Parandin *et al.*, 2016
	**↑**	Tamoxifen	1 dose, PND1	100 µg	3.5 months (PND106)	Oestrus days (average, in 10 examination days)	*In vivo*	Rat (Wistar)	Csaba and Karabelyos, 2001
**Sexual behaviour**	**↓**	Tamoxifen	5 days, PN1–5	12.5 and 100 µg	PND243	Adult males (mount, intromission and ejaculation latency)	*In vivo*	Rat (Wistar)	Morales-Otal *et al.*, 2005
	**↓**		1 dose, PND1	100 µg	3.5 months (PND106)	Female sexual activity (lordotic response) and male sexual activity (mounting)	*In vivo*	Rat (Wistar)	Csaba and Karabelyos, 2001
	**↑**	Letrozole	3 days, GD16-18	1.25 mg/kg (vs 1 mg/kg and control groups)	PND60	Males—mounting, intromission and ejaculation	*In vivo*	Rat (Sprague- Dawley)	Shaaban *et al.*, 2023

Up/down arrows denote significant differences compared to control groups (*P*<0.05). The horizontal bidirectional arrow denotes no significant difference. E, embryonic day; GD, gestational day; dpp, days post-partum; FSH, follicle-stimulating hormone; PND, postnatal day.

A total of 13 studies examined the foetal reproductive impact of hormonal drug exposure. All studies used rodent models with exposure to either tamoxifen or letrozole. These studies and effects reported are summarized in Table 3. Tamoxifen exposure significantly reduced the number of germ cells in the testis after a single dose (Gonzalez-Gonzalez *et al.*, 2017), with similar effects found in the ovary following both single and repeated tamoxifen exposures (Razvi *et al.*, 2007; Roshangar *et al.*, 2010; Zhao *et al.*, 2023). Likewise, letrozole exposure also reduced germ cell number in rat foetal testes, resulting in a significant reduction in spermatogenesis in adulthood (Shaaban *et al.*, 2023). In addition, testosterone levels in rat male offspring were reported to have significantly increased at PND60, after three doses at varying concentrations given between gestational Days 16 and 18 *in vivo*, resulting in an increased anogenital distance (Shaaban *et al.*, 2023). Studies examining other cell types within the testis found a reduction in Sertoli cell number following a single tamoxifen dose at birth, but no impact on Leydig cell number (Gonzalez-Gonzalez *et al.*, 2017). Both tamoxifen and letrozole caused disruption of the testicular architecture in rats (Gonzalez-Gonzalez *et al.*, 2017; Shaaban *et al.*, 2023).

Detrimental effects were also found in hormone therapy-exposed foetal ovaries, where a significant increase in apoptosis, DNA double-strand breaks, and epigenetic modifications were reported in mouse ovaries after a single *in utero* tamoxifen dose (Zhao *et al.*, 2023). A significant decrease in PMF numbers and corpora lutea was observed following foetal exposure to tamoxifen *in vivo* (Razvi *et al.*, 2007; Zhao *et al.*, 2023). Interestingly, the number of germ cell nests was significantly increased in the mouse ovary after foetal tamoxifen exposure (Roshangar *et al.*, 2010). Uterine and ovarian weights were also significantly reduced after tamoxifen exposure in mice (Hilakivi-Clarke *et al.*, 2000; Karlsson, 2006; Razvi *et al.*, 2007; Parandin *et al.*, 2016).

## Discussion

To the best of our knowledge, this is the first systematic review evaluating the evidence for the impact of *in utero* exposure to cancer treatments on foetal reproductive development and the potential implications for subsequent reproductive function and fertility. To date, studies investigating the adverse effects of *in utero* chemotherapy exposure on the offspring have primarily focused on postnatal outcomes such as neurological and cardiac effects or on major congenital malformations (Murthy *et al.*, 2014; de Haan *et al.*, 2018; Safi *et al.*, 2019; Blommaert *et al.*, 2020), but have not reported on reproductive outcomes of the individuals who were exposed to cancer treatments *in utero.* The studies included in our systematic review demonstrate evidence of the significant vulnerability of foetal reproductive development to chemotherapy and hormone therapy exposure.

### Role of the placenta

**Figure 3.**
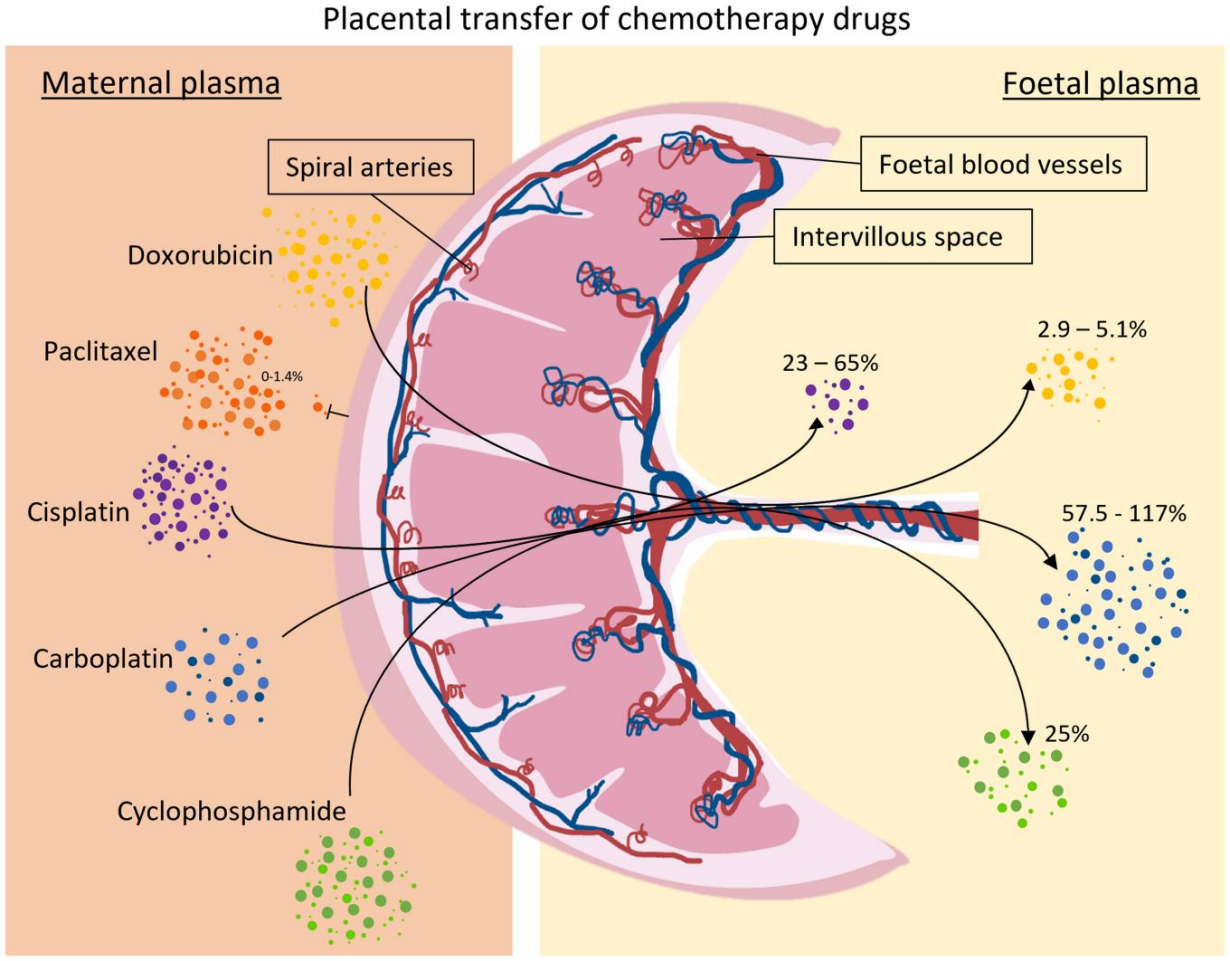
**Schematic to demonstrate the variation in placental perfusion of different chemotherapy drugs.** Data from mouse, baboon, and human models of commonly used chemotherapies in pregnant cancer patients that pass through the placenta. Percentage in foetal compartment as compared to maternal plasma concentration (Benoit *et al.*, 2021; Van Calsteren and Amant, 2011).

One of the key considerations for any study examining the impact of *in utero* exposure to cancer treatments is understanding their rate of placental transfer and the level of exposure for the foetus. Available experimental placental perfusion studies have demonstrated that chemotherapy drugs are able to pass through the placenta and enter the foetal blood stream (Benoit *et al.*, 2021; Triarico *et al.*, 2022). One of the chemotherapy drugs examined in several of the studies in this review, cisplatin, has a low molecular weight but a high capacity to bind to plasma proteins (Van Calsteren *et al.*, 2010). Whilst few studies have explored the transplacental transfer of cisplatin, one study using a human placenta found cisplatin concentrations as high as 42% and 65% of that of the maternal blood in the amniotic fluid and umbilical artery, respectively (Köhler *et al.*, 2015), suggesting that cisplatin may be one of the chemotherapy agents with the highest rate of transplacental transfer. In comparison, cyclophosphamide showed a 25% foetal transfer rate in a baboon model of placental transfer, and 25% of maternal plasma drug levels were detected in the amniotic fluid of an *in vivo* human pregnancy (Van Calsteren and Amant, 2011). Paclitaxel showed no evidence of placental transfer and very low (1.5%) transfer in mouse and baboon placental models, respectively, but was detected in neonates’ meconium (Benoit *et al.*, 2021). The known placental transfer rates and detections in the foetal compartment are illustrated in Fig. 3, demonstrating the variation in potential exposure of the foetus to chemotherapy administered to the pregnant mother.

### Impact of chemotherapy and hormone therapy agents on foetal reproductive development

The data gathered from the studies included in this review demonstrate the foetal gonadotoxicity of a number of chemotherapies and hormone therapies. The most consistent effect observed in the gonads of both sexes, across several studies, models, and drug types, was the detrimental impact found on germ cell number. Furthermore, in females, a reduction in the number of PMFs formed within the ovary, and in some cases, premature activation of these follicles, was observed in some of the studies. This could be associated with a diminished follicle pool, early menopause, or premature ovarian insufficiency (POI). Indeed, mice prenatally exposed to the chemotherapy drugs docetaxel and paclitaxel had fewer litters overall, with the mice in the paclitaxel group also having their last litter earlier (Chaqour *et al.*, 2024), potentially reflecting POI. Moreover, loss of ovarian activity might not only result in sub- or infertility but also persistent oestrogen deficiency can affect bone function, cardiovascular, and neurological health (Spears *et al.*, 2019). Exposure to tamoxifen reduced both ovarian and uterine weight, whilst increasing the number of germ cell nests within the ovaries and reducing the number of PMFs formed. These results are consistent with the oestrogenic activity of tamoxifen, which binds to ERs and produces both oestrogenic and anti-oestrogenic effects (Farrar and Jacobs, 2023). Interestingly, mice exposed to the chemotherapy agent docetaxel *in utero* were also found to have an increased number of multi-oocyte follicles in their ovaries (Chaqour *et al.*, 2024). Indeed, numerous studies have demonstrated that exposure of the developing foetal ovary to oestrogenic compounds disrupts cyst breakdown, giving rise to multiple oocyte follicles whilst reducing the number of PMFs formed and reducing the size of the follicle pool (Jefferson *et al.*, 2002, 2006; Chen *et al.*, 2007, 2009; Pepling, 2012).

Chemotherapy drugs and hormone therapy agents resulted in reduced testicular weight in all studies reporting on this outcome, apart from one. Furthermore, the studies examining the effects of chemotherapy exposure on human foetal testicular tissue suggest that the timing of exposure within the second trimester could be of particular importance, where germ cell numbers in the early second trimester testis were not impacted by treatment, but with a detrimental effect found in testicular tissue from later stages of the second trimester (Matilionyte *et al.*, 2022a). This suggests that the human foetal testis might be more vulnerable to chemotherapy exposure in the later stages of the second trimester. This could be due to differences in the rate of proliferation in testicular cell (sub)types, rendering them more vulnerable at certain time points. During the second and third trimesters, there is a gradual transition of the germ cell population from gonocytes to (pre)spermatogonia. The changes in germ cell sensitivity to cisplatin during the second trimester may be due to the variation in the proliferation rate between these germ cell sub-populations and the changes in proliferation rate in individual germ cell populations over time (Mitchell *et al.*, 2010; Matilionyte *et al.*, 2022a). Given that many of the key gonadal cell types are undergoing consistent and rapid proliferation in foetal life, it is possible that cancer treatments that are designed to target rapidly dividing cells could have a detrimental effect on the proliferation of these cells during gonadal development. Indeed, the germ cells in both the ovary and testis go through rapid phases of proliferation, which render them more vulnerable to the types of chemotherapy drugs that specifically target rapidly proliferating cells (Tharmalingam *et al.*, 2020; Matilionyte *et al.*, 2022a, 2023). However, the gonadotoxicity of the drugs was not only confined to the germ cells, where both Leydig- and Sertoli cells of the foetal testis were shown to be detrimentally affected by chemotherapy agents and hormone therapy treatments, respectively. Nevertheless, loss of either the spermatogonial stem cells or the supporting somatic cells can have a detrimental impact on long-term fertility.

The hypothalamic–gonadal–pituitary axis is established during foetal life, and consequent disruption to this, via chemotherapy or hormonal therapies, could lead to detrimental effects on fertility in later life. There appears to be a consistent disruption to the cellular functions of the testis, causing changes to the organ weight and hormone production, both of which could have long-lasting impact into adulthood. Evidence from both human and animal studies has shown that disruption to the development of the reproductive system during foetal life can result in the onset of reproductive disorders in later life, such as testicular dysgenesis syndrome (TDS) in males and endometriosis or premature ovarian failure in females (Rhind *et al.*, 2001; Main *et al.*, 2006; Monniaux, 2018). Indeed, exposure to gonadotoxic and/or oestrogenic compounds during the MPW, which occurs between E15.5–18.5 in rats, and estimated to be 8–14 weeks of human gestation, and a subsequent impairment of androgen production in the foetal testis, has been strongly associated with TDS in later life (Sharpe, 2020). The exact mechanism of TDS is unknown, but suppression of testosterone production, androgen receptor expression, or Leydig cell function in the foetal testis has been associated with subsequent reproductive impacts in adulthood, including cryptorchidism, hypospadias, dysfunctional testis, as well as testicular cancer and impacts on fertility (Skakkebaek *et al.*, 2001; Sharpe, 2003). In this systematic review, only two studies were identified that reported the impact of chemotherapy drugs on Leydig cell number and testosterone levels, with both of these drugs, cytarabine and busulfan, showing a detrimental effect on these reproductive outcomes; importantly, the exposure windows in both studies overlapped with the MPW (Hall and Gomes, 1973; Namoju and Chilaka, 2022). For hormonal cancer treatments, only one study explored the impact of tamoxifen on Leydig cell number, reporting no detrimental impact (Gonzalez-Gonzalez *et al.*, 2017). Given the distinct lack of data on the impact of cancer treatments on Leydig cells and/or testosterone production, combined with the lack of long-term follow-up studies on reproductive health, there remains a distinct gap in knowledge about how these cancer treatments might be impacting the reproductive health of males exposed *in utero*. This is particularly pertinent given the well-known oestrogenic effects of tamoxifen (Farrar and Jacobs, 2023).

Whilst the majority of the studies investigated effects on the foetal reproductive systems, it is important to consider the possibility of a transgenerational ‘grand-maternal effect’ if the drug elicits genetic damage in the exposed foetal germ cells that could be passed on to subsequent generations. One study demonstrated multi-generational effects on ovarian health, where the female offspring of female mice that were exposed to docetaxel *in utero* (E16.5) had increased levels of atretic secondary follicles (Chaqour *et al.*, 2024). Many chemotherapy drugs, including cisplatin, doxorubicin, etoposide, and cyclophosphamide, act by inducing double-stranded DNA breaks within the cancer cell, activating the apoptotic machinery (Halim *et al.*, 2018; Wang *et al.*, 2018; Gold and Raja, 2023; Yadav *et al.*, 2023). In the studies reviewed here, there was evidence of increased levels of cellular apoptosis (albeit contradicting) and oxidative stress in chemotherapy-treated gonads (Tharmalingam *et al.*, 2020; Matilionyte *et al.*, 2022b; Namoju and Chilaka, 2022; Chaqour *et al.*, 2024), as well as increased levels of DNA double-stranded breaks and epigenetic effects following tamoxifen exposure (Zhao *et al.*, 2023). It is important to note that not all germ cells suffering genetic insult undergo apoptosis, which means that in the ovary, this could result in oocytes progressing through meiosis despite the presence of DNA damage (Marangos *et al.*, 2015). It is theoretically possible, therefore, that damaged germ cells could go on to form follicles, ovulate, and potentially result, for example, in an aneuploid embryo in the second generation, which would have devastating consequences for both the parents and the offspring.

Taken together, there is a distinct possibility that the foetal gonad could be vulnerable to cytotoxic insult during maternal chemotherapy and/or hormone treatment, potentially affecting future fertility. Delayed clinical recognition is very likely and might not present until adolescence, while more subtle effects on fertility would not be evident until much later, potentially when the person wishes to have a child themselves or if they enter early menopause.

### Limitations

This systematic search looked for all published documents in a range of databases and provides a comprehensive overview of the evidence at the time for the effects of *in utero* chemotherapy exposure. However, there is inevitable variability in quality between the different studies included in this review, further complicated by an inability to test the robustness of each of the findings. There will also be inherent publication bias in our findings, with the publication process often favouring studies reporting significant experimental findings over those reporting negative findings. In addition, we have been unable to group and compare the data presented by each study to perform a meta-analysis, due to the heterogeneity of the results, with the studies using differing doses, drugs, and animal models.

Findings from the animal model studies cannot be directly extrapolated to humans, due partly to interspecies differences in metabolism and placental anatomy, as well as the fact that rodents are polyovular, delivering litters, while humans are mono-ovular, most often having a singleton pregnancy. Given the limitations in extrapolating findings from rodent models, further research will be needed in other reproductive developmental study models or, ideally, in follow-up studies on the offspring of the pregnant cancer patients themselves. For the purpose of this review, a decision was made to include all studies that covered any gestational stage, including any studies up to and including PND4 in the rodent models. This is because that particular time window has been found to be more closely aligned to the developmental processes and stages of pre-natal human gonadal development (Hartshorne *et al.*, 2009; Allen *et al.*, 2020). Similarly, the *in vitro*-based exposure studies will be limited in their application for understanding the actual reproductive impact of chemotherapy, as they do not replicate exposures *in utero* or longer-term effects to foetal tissue that may be seen following exposure. Moreover, experimental studies often focus on the short-term effects on cells and tissues and do not look at the long-term impacts, such as sperm production, fertility, or potential transgenerational effects.

We have been unable to identify any clinical cohort studies analysing reproductive outcomes among those exposed to cancer treatment *in utero*. The human studies identified perform *in vitro* exposure of foetal tissues, which limits extrapolation of these results more widely but does give valuable insight into the effects of chemotherapy agents on human foetal gonadal tissues. Moreover, as previously mentioned, chemotherapy and hormone therapy drugs are usually administered in a combination of drug classes and regimens. This is not reflected in any of the studies included in this review, where all the studies investigated single chemotherapy or hormonal therapy exposures. In addition, the doses used in experimental studies may not accurately reflect real-life foetal exposure levels. Consequently, there are currently no available data on the impact that a cocktail of drugs administered to a pregnant cancer patient may have beyond those seen in the included studies.

### Future implications

The results of this systematic review emphasize the distinct lack of published research investigating the impact of anticancer agents on foetal reproductive development *in utero*. The majority of the available data come from animal models, with only 4 out of the 26 studies included in the review having been conducted on human tissues, specifically the human foetal testis. As of yet, no published studies have examined the impact of cancer treatments on human foetal ovarian tissue. The remainder of the studies included in the review were carried out on mouse and rat models, which, whilst providing an important basis for beginning to understand the potential detrimental effects of *in utero* exposure to cancer therapies on gonadal development, highlight the lack of available data in this area. Hormonal therapy is now contraindicated during pregnancy; therefore, the priority should be to evaluate the impact of chemotherapy agents on the developing foetal gonads and on the future reproductive health of individuals exposed *in utero*. Work should focus on those drugs considered acceptable to administer to pregnant cancer patients and commonly used in that patient group (Esposito *et al.*, 2016). The currently available follow-up studies on children who have been exposed to chemotherapy drugs *in utero* have not examined or reported on reproductive outcomes (Greiber *et al.*, 2022; Huis In ’t Veld *et al.*, 2024). As a consequence, the lack of available data on reproductive impact could potentially result in undiagnosed sub- or infertility. Counselling on the potential adverse reproductive effects of chemotherapy treatment is often prioritized for adult or child cancer patients, but this is likely not the case for those born after *in utero* exposure to the same gonadotoxic treatments. Nevertheless, reproductive health is an important aspect of an individual’s long-term health and wellbeing and is the top non-survival-related concern among reproductive-age women who face cancer treatment (Xie *et al.*, 2022). The long-term reproductive health of *in utero*-exposed children should therefore be taken into consideration by healthcare providers and researchers.

A diagnosis of cancer during pregnancy may be further complicated in certain countries where abortion is not legal, including in parts of the USA, where recent changes in abortion laws following the overturning of Roe. v. Wade has led to increased ethical and legal concerns surrounding the treatment of pregnant women with cancer (Arup and Shravan, 2023). In these places, restricted access to abortion due to medical reasons, such as cancer treatment, where the patient will urgently require treatment, will further complicate an already challenging diagnosis and treatment plan, potentially leading to a further rise in the number of children being exposed to cancer treatments *in utero.*

By carrying out this systematic review, we have demonstrated the distinct lack of clinical studies that have explored this effect, yet all the relevant studies included in this review point towards a detrimental impact on reproductive development in both sexes following *in utero* exposure to cancer treatments. With this in mind, it is clear that further research on appropriate animal models and human tissues must be conducted in order to better understand the impact on future fertility. Moreover, current long-term studies investigating the outcomes of individuals exposed *in utero* (Greiber *et al.*, 2022; Huis In ’t Veld *et al.*, 2024) should place fertility among the key indicators of longer-term health and wellbeing measures in their *in utero*-exposed cohort. Without these data, we remain unaware of any potential cohort findings on reproductive health and fertility outcomes. Understanding this impact would enable more accurate information to be given to those deciding on cancer treatments during pregnancy and would also enable those who have already been exposed to chemotherapy *in utero* to access timely fertility care and potential preservation treatments if this is required, such as cryopreservation of oocytes, testicular tissue, or sperm (Chen *et al.*, 2023).

## Conclusion

The evidence presented here from both *in vitro* and *in vivo* studies of exposure to chemotherapy and hormone therapy in developing gonads and other reproductive tissues, in both rodent models and human foetal tissue, points towards the possibility of adverse reproductive development and fertility as a result of exposure. The most consistent effect observed in the gonads of both sexes, across several studies, models, and drug types, was the detrimental impact found on germ cell number. Exposure to tamoxifen reduced both ovarian and uterine weight whilst increasing the number of germ cell nests within the ovaries and reducing the number of PMFs formed. The results of this systematic review also emphasize the distinct lack of published clinical research investigating the impact of anticancer agents on foetal reproductive development *in utero*, both in the short and long term. Taken together, our data demonstrate the real probability that the foetal gonad could be vulnerable to cytotoxic insult during maternal chemotherapy and/or hormone treatment, potentially affecting future fertility.

Ultimately, improved understanding in this area will offer clinicians and pregnant cancer patients accurate and up-to-date information about the impact of chemotherapy treatment on the foetal reproductive system to inform decisions and mitigate risk to the foetus and mother. Moreover, we emphasize the importance of following up and assessing key reproductive indicators in children and young adults who have been exposed to cancer treatments *in utero*.

## Supplementary Material

hoaf046_Supplementary_Data

## Data Availability

The data underlying this article are available in the article and in its online supplementary material.
