# Supplementary material for: The impact of in utero exposure to cancer treatments on foetal reproductive development and future fertility: a systematic review
Source: Hum Reprod Open. 2025 Jul 23;2025(3):hoaf046. doi: 10.1093/hropen/hoaf046 (PMC12366489; doi:10.1093/hropen/hoaf046)
Supplement: hoaf046_Supplementary_Data [file hoaf046_supplementary_data.zip › hoaf046 Supplementary Tables.docx]

**Supplementary Table S1. Key characteristics and risk of bias scores for each included study in this systematic review.**

| Num-ber | Study ID (author/year) | Study design | Methods | Chemo-therapy drug/class | Aim(s) | Test indicators | SciRAP (risk of bias) score – *in vivo* or *in vitro* | | Reliability category* |
| --- | --- | --- | --- | --- | --- | --- | --- | --- | --- |
|  |  |  |  |  |  |  | Reporting | Methods |  |
| 1. Animal-model studies | | | | | | | | |  |
| 1 | Namoju and Chilaka, 2022 | *In vivo* | Pregnant rats (strain not stated) received intraperitoneal treatment with Cytarabine (Ara-C): 12.5 mg/kg or 25 mg/kg doses. All male pups were collected on PND1 (day of parturition). Estimation of oxidative stress protein markers (unclear which method was used), ELISA for fetal testosterone levels, histopathology and morphometry. | Cytarabine (Ara-C) | To explore the benefits of maternal supplementation of Alpha-lipoic acid against *in-utero* Ara-C exposure-induced testicular toxicity in rat fetuses. | Foetal outcome parameters evaluated bodyweight, anogenital distances, testicular weight, testicular testosterone levels, testicular histopathology and morphometrical parameters. | 81.67 | 85.29 | 1 |
| 2 | Zhao et al., 2023 | *In vivo* | Oral administration of Tx. Immunohistochemistry, Immunofluorescence and immunocytofluorescence. Western blots. qRT-PCR. TUNEL stain. ELISA. Edu Cell proliferation kit. | Tamoxifen (Tx): antiestrogen | To explore the effects of maternal tamoxifen exposure on infants in utero, using the mouse model. | Day 3 ovaries: Immunofluorescence for markers of germ cells (MVH) and primordial follicle assembly (LHX8 and SOHLH1). mRNA levels of Lhx8, Figla, Sohlh2 were examined by RT-PCR. Day 21 ovaries: Follicle counts and western blotting for genes related to follicular development (BMP15 and GDF9). Granulosa cell proliferation was assessed with a cell proliferation kit. TUNEL staining and DNA double strand breaks. Histone methylation studied. | 53 | 61 | 2 |
| 3 | Comish et al., 2014 | *In vivo* | Intra-perineal (I.P.) injection of pregnant dams. H&E staining of tissue sections. | Cyclophosphamide: alkylating agent | To determine whether fetal exposure to cyclophosphamide induces testicular cancer and/or gonadal toxicity in 129 and in 129.MOLF congenic (L1) mice. | In testis: incidence of testicular germ cell tumours, testis weight, number of testes with atrophic tubules, testicular and epididymal sperm counts. In ovary: follicle 4counts (primordial, primary-transitional, p5rimary, secondary). | 68 | 86 | 1 |
| 4 | Parandin et al., 2017 | *In vivo* | Subcutaneous injection of newborn mice for 5 days with Tx. Animals culled on PND70. Observation of reproductive outcomes in these exposed mice. | Tamoxifen (Tx): antiestrogen | To evaluate whether neonatal exposure to tamoxifen affects oestrogenic actions in the brain and reproductive function in mice. | Time of vaginal opening, oestrous cycle and follicle pool. Kisspeptin mRNA expression, neuronal density of AVPV and ARC nuclei and LH and oestradiol concentrations in serum. | 75 | 94 | 1 |
| 5 | Gonzalez-Gonzalez et al., 2017 | *In vivo* | Newborn male rats received a single dose of Tx, or SO one hour after birth and returned to mothers until weaning. Animals were culled 90 days post treatment for morphological assessment of testes and testis weight | Tamoxifen (Tx): antiestrogen | To evaluate the effects of administration of tamoxifen and its vehicle, soybean oil, during the critical period of hypothalamic sexual differentiation in newborn male rats in the context of gonadal histomorphology during adulthood. | Lower testicular weight in Tx group compared with control group and SO-treated group. Tx group also had sig reduction in tube diameter, area, and height of germinal epithelium. Tx also increased the intertubular space. Sig reduction in number of spermatogonia and Sertoli cells in Tx and SO groups compared to control. No diff in number of Leydig cells. | 66 | 88 | 1 |
| 6 | Nakai et al., 1999 | *In vivo* | Male pups received daily subcutaneous injections of Tx (2 ug) from PND1 to PND5 (day of birth = PND0). Males were killed at around 3 months of age. | Tamoxifen (Tx): antiestrogen | To determine if abnormalities to the male reproductive system after neonatal exposure to tamoxifen are induced in a mouse strain specific manner. | Body weight, testicular weight, gross malformation, histopathology of the testis, epididymis, ductus deferens and seminal vesicles | 45 | 82 | 1 |
| 7 | Morales-Otal et al., 2005 | *In vivo* | Male pups received daily subcutaneous injections of Tx (100 ug per 0.05 of corn oil) or vehicle only (corn oil) between days 1-5 after birth. Males were kept until 5-6 months of age. | Tamoxifen (Tx): antiestrogen | To determine if perinatal administration of Tx affects brain sexual differentiation through a permanent inhibitory action on the fetal testicular secretion of testosterone. | Hormonal assessment, histological analysis of reproductive organs (testis) | 56 | 94 | 1 |
| 8 | Karlsson, 2006 | *In vivo* | Tx exposure of newborn male and female Sprague-Dawley rats (days 1–5) to investigate the occurrence of developmental abnormalities in the adulthood. At the age of 15 months, the animals were necropsied, and organs were collected for histopathology and histomorphometry. | Tamoxifen (Tx): antiestrogen | To describe histopathological effects in male and female rats treated neonatally, with an emphasis on toremifene. The effects are compared to those produced by oestrogens. | Histological examination of reproductive organs. | 63 | 88 | 1 |
| 9 | Roshangar et al., 2010 | *In vivo* | i.p. injection of pregnant female mice. Reproductive tract was removed from pups between days 2-7 after birth. Ovaries were examined histologically | Tamoxifen (Tx): antiestrogen | To evaluate the effect of tamoxifen on oocyte and follicular development and differentiation in mice. | Reproductive tracts were removed from pups at 2, 3, 6 and 7 days old (8 mice per group per age) and ovaries dissected away for histological analysis. | 50 | 64 | 3 |
| 10 | Csaba and Karabelyos, 2001 | *In vivo* | Newborn (within 24 h after birth) male and female rats were treated with a single dose of 100 μg mifepristone or 100 μg tamoxifen, subcutaneously. Controls received the vehicle. When the animals reached adulthood (three and a half months old) their sexual behavior was tested. | Tamoxifen (Tx): antiestrogen | To understand the effect of neonatal treatment with tamoxifen or mifepristone on the sexual behaviour of adult male and female rats. | Sexual behaviours: receptivity of female rats to males: lordosis quotient and Meyerson index screened during oestrus. Male mounting behaviour, seeking behaviour for 5 mins, | 45 | 70 | 2 |
| 11 | Malekinejad et al., 2011 | *In vivo* | Fetuses on gestation day 20, and from PND 5 were fixed and stained for investigation of gonads. The entire fetus samples and the ovaries from the neonates were fixed in the 10% buffer phosphate formaldehyde solution. number of different types of follicle were compared by using one-way ANOVA test. | Tamoxifen (Tx): antiestrogen | The effect of tamoxifen as a selective estrogenic receptor modulator on the folliculogenesis in rat's foetuses and neonates. | Histological analysis of the fetal genital tracts, number of follicles formed in ovaries. Morphology pf the gonads. | 66 | 82 | 1 |
| 12 | Hall and Gomes, 1973 | *In vivo* | Day 13, each female was injected intraperitoneally with busulphan (10 mg/kg body wt) or with the vehicle (arachis oil) alone and allowed to litter. At 21, 40 and 60 days after birth, male offspring were weighed and exsanguinated by cardiac puncture under sodium pentobarbital anaesthesia. The seminal vesicles, except in the 21 day-old group and the testes were removed and weighed. Testis samples taken for histological examination were fixed in Bouin's solution, embedded in paraffin wax, and sectioned. H&E staining. testis tissue was weighed, homogenized in distilled water and stored. Serum was separated from clotted blood samples and also stored at —20° C. Testosterone levels were assayed in ether extracts of serum and tissue homogenates using radioimmunoassay. | Busulphan | To determine the testosterone levels in the testes and serum of rats exposed prenatally to busulphan. | Testis and seminal vesicle weights. Microscopic examination of the testis. Concentration of testosterone. | 35 | 67 | 2 |
| 13 | Ray and Potu, 2010^#^ | *In vivo* | Pregnant rats (n=12) were randomly assigned cyclophamide or control groups. Sacrificed gestation day 20 and fetuses collected. Stained with H&E, for observation of the ovaries. | Cyclophosphamide: alkylating agent | To investigate whether cyclophosphamide interferes with ovarian folliculogenesis. | Examination of offspring ovaries morphology. | 55 | 56 | 3 |
| 14 | Stefansdottir et al., 2016 | *In vitro* | Culture of fetal and newborn mouse ovaries, H&E staining for histological analysis. Immunohistochemistry for markers of meiosis (sycp3) and TopoIIa. | Etoposide: topoisomerase II inhibitor | To use ovarian tissue culture methods to investigate the effect of etoposide on germ cells prior to and following follicle formation. | Follicle counts (primordial/transitional/primary/secondary). IHC analysis of meiotic progression through sycp3 staining. | 71 | 83 | 1 |
| 15 | Razvi et al., 2007 | *In vivo* | subcutaneous injection of mice with vehicle or tx. Uteri, ovaries and other major organs were collected and examined. Uteri were weighed. Uteri and ovaries were fixed. | Tamoxifen (Tx): antiestrogen | The effects of subcutaneous dosing of neonatal CD-1 [mice](https://www.sciencedirect.com/topics/veterinary-science-and-veterinary-medicine/mouse) with [tamoxifen](https://www.sciencedirect.com/topics/pharmacology-toxicology-and-pharmaceutical-science/tamoxifen) on days 1–5 after birth at doses of 0, 5, 10, 25 or 50 μg/pup or with 4-hydroxyoestradiol at 2 μg/pup have been investigated. | Uteri, ovaries and other major organs were collected and examined. Uteri were weighed. Uteri and ovaries were fixed. | 50 | 73 | 2 |
| 16 | Shaaban et al., 2023 | *In vivo* | Pregnant rats randomly assigned into 5 experimental groups (n = 3/each) and orally received either letrozole at doses of 0.25, 0.75, 1.00, and 1.25 mg/kg body weight (BW) or vehicle (control) on the gestation days of 16, 17, and 18. Pregnancy outcome, sexual behaviours on postnatal day 60, serum biochemical features, and the histopathology of testes were assessed in male offspring testes and epididymides were removed, weighed, and fixed in 10% buffer formalin for histological evaluation. | Letrozole (aromatase inhibitor) | To assess the effects of in utero exposure to letrozole and its late consequences on the reproductive and metabolic performance of adult male offspring. | Sexual behaviours, morphology of testes and epididymis. | 70 | 85 | 1 |
| 17 | Green et al., 2005 | *In vitro* | Pdfgra, Sox9, AMH and Foxl2 expression measured by whole-mount in situ hybridization and Q-PCR. Protein levels of FOXL2, AMH and SOX9 measured by IHC. In vitro study: Gonads dissected from E13.5 and cultured for 3 days with or without Tx. Expression of Pdgfra, Jag1 and Fgf9 in cultured gonads were analysed by Q-PCR. Culture media was collected for radioimmunoassay to measure testosterone, progesterone and E2 levels. | Tamoxifen(Tx): antiestrogen | To examine the effects of short-term tamoxifen exposure in neonate mice in order to correlate long- term changes in uterine pathology and gene expression. | Uteri were weighed. Ovaries, uteri and other major organs were histopathologically examined. Uterine sections were stained with van Gieson's stain for collagen. Uteri snap frozen for RNA extraction. IHC analysis for estrogen receptors and Ki-67. cDNA microarrays were carried out on uteri | 50 | 70 | 2 |
| 18 | Clarke et al., 2000 | *In vivo* | Administration of tamoxifen to pregnant rats at days 15-20 pregnancy or with control. Pups examined at day 21 for uterine weights, and for day of vaginal opening. Statistical analysis comparing control and treated groups to look for effect of tamoxifen exposure *in utero*. | Tamoxifen (Tx): antiestrogen | to determine whether tamoxifen acts as an estrogen in the fetal mammary gland and increases breast cancer risk. | Main outcomes of the study were mammary tumorigenesis - reproductive outcomes were reported as part of the study. Uterine weight compared to controls, puberty onset (day of vaginal opening), | 65 | 73 | 2 |
| 19 | Ozcan et al., 2023 | *In vitro* | Neonatal ovaries were dissected with tissue forceps and syringe needle in sterilized phosphate-buffered saline (PBS) under a dissecting microscope under sterile conditions. Ovaries were cultured in 24-hanging-well-culture plates. cyclophosphamide, doxorubicin, docetaxel, paclitaxel, and cisplatin exposures. The chemotherapy agent or control DMSO was added to the culture in a single exposure at 48 h or postnatal day 2. Media were then changed every 48 h thereafter. Whole ovaries collected for analysis PND 4 or PND7. Immunoflourescence staining. | cyclophosphamide, doxorubicin, docetaxel, paclitaxel, and cisplatin | Understand effects of chemotherapy exposure in early postnatal oocytes and ovarian reserve in ex vivo mouse model. | Tissue staining - reproductive organs. Oocyte density at PND4 and PND7 following culture. Cell death staining and analysis. | 64 | 83 | 1 |
| 20 | Lopes et al., 2014 | *In vitro* | Newborn mouse ovaries were dissected and placed in culture in 24-well plates. Medium was supplemented on Day 2 with low, medium or high docetaxel (or 0.1% ethanol vehicle for control) concentrations and moved into fresh control medium on day 3 and cultured until day 6. Half the media was replaced on Day 5. Whole ovaries after 6 days in culture were fixed, sectioned and analysed by histological and immunohistochemical analysis. | Docetaxel | To investigate docetaxel-induced damage to the resting pool of primordial follicles and to the early stages of follicular development. | Main outcomes were a non-significant decrease in follicle number in the high docetaxel group, although a significant decrease in the number of primary follicles. There was also a significant dose-dependent increase in % of unhealthy follicles, with the % of unhealthy transitional and primary follicles increasing at all doses tested. There was no increase in the % of unhealthy primordial follicles across treatment groups. Docetaxel also increased the % of the apoptotic markers CC3 and CC8 positive area and CC3 positive follicles in all treatment groups. Docetaxel also increased Bax and cPARP protein levels in ovaries exposed to the high dose of docetaxel, whilst decreasing the protein levels of CytC. | 72.5 | 83.33 | 1 |
| 21 | Morgan et al., 2013 | *In vitro* | Newborn female mouse ovaries cultured in 24-well plates, with media supplemented with different doses of cisplatin or doxorubicin-HCl on day 1 of culture. Ovaries were fixed either immediately after drug treatment (Day 2) or moved into drug free media for Days 3-6. 50% of the medium was changed every other day. Ovaries were snap frozen for protein extraction and Western Blotting, or fixed for TUNEL analysis or histological analysis. | Cisplatin  Doxorubicin | To investigate mechanisms by which cisplatin and doxorubicin cause follicle loss using an in vitro system to culture mouse ovaries. | Main outcomes were a significant increase in the percentage of unhealthy follicles following both cisplatin and doxorubicin exposure, and a significant decrease in the number of follicles (cis at highest dose only, dox at all but lowest dose). There was an increase in the % of unhealthy transitional and primary follicles after treatment with both drugs. There was an increased expression of cleaved PARP after treatment with both drugs, but no change in TUNEL staining in oocytes or granulosa cells. | 60 | 83.33 | 1 |
| 22 | Chaqour et al., 2024 | *In vivo* | Pregnant dams treated with single dose of saline (control), docetaxel or paclitaxel at embryonic day 16.5. ovarian (H&E staining) and endocrine analysis of the female offspring at multiple time points (14 and 30 days after birth) and also bred to assess fertility. Offspring of exposed females also assessed for ovarian follicle composition and death. | Docetaxel (taxane) and paclitaxel (taxane) | To investigate the effects of taxane-based chemotherapy on the developing foetal ovary. | Ovarian follicle composition, rates of follicle atresia, and rates of multioocyte follicles in all exposure groups. Serum hormone levels and oocyte retrieval outcomes following ovarian hyperstimulation. Offspring from all exposure groups were bred and the number of litters, pups per litter, live births, interlitter time interval, and age at the last litter analysed. | 55.00 | 70.59 | 2 |
| *2. Human-based studies* | | | | | | | | |  |
| 23 | Matilionyte et al., 2023 | *In vitro* | in vitro culture of human and mouse foetal testis tissue with exposure to differing concentrations of cisplatin. Hanging drop culture of human testis, cisplatin or control on day 3 of culture for 24 hours. Tissue culture for 14 days after exposure. immuno-staining of tissue sections from each treatment, with positive and negative controls. staining to detect germ cells/gonocytes and pre-spermatogonia. Statistical analysis to compare treatment groups and controls. Single-RNA sequencing for human testis to define cell populations. | Cisplatin (Platinum-based alkylating agent) | To investigate whether exposure to one of several regimens of combined granulocyte-colony stimulating factor and cisplatin could protect the key germ cell populations from cisplatin-induced damage in immature human testicular tissues, and to identify species-dependent effects of G-CSF exposure with and without cisplatin by comparing findings in human and mouse immature testicular tissues. | Immunostaining for gonocytes and pre spermatogonia. Staining to detect germ cells and SSC sub-population in mice. RNA sequencing of human fetal and pre-pubertal testis. to define the cell populations present in the testis and to localise expression of CSF3R. | 76 | 93 | 1 |
| 24 | Matilionyte et al., 2022a | *In vitro* | in vitro culture system to culture pieces of human fetal testicular tissues (total n=23 fetuses) from three different gestational age groups (14-16 (early), 17-19 (mid) and 20-22 (late) gestational weeks; GW) of the second trimester. Tissues were exposed to cisplatin or vehicle control for 24 hours, analysing the tissues 72 and 240 hours post- exposure. Number of germ cells and their sub-populations, including gonocytes and (pre) spermatogonia, were quantified. | Cisplatin (Platinum-based alkylating agent) | To determine whether effects of exposure to cisplatin on the germ cell sub- populations are dependent on the gestational age of the foetus and what impact this might have on the utility of using human foetal testis cultures to model chemotherapy exposure in childhood testis. | Immunostaining and cell quantification - germ cells (gonoctyes and pre-spermatogonia cells). | 76 | 93 | 1 |
| 25 | Tharmalingham et al., 2020 | *In vitro* | Organotypic culture system with xenografting onto host mice per sample. Human foetal and prepubertal testicular tissues were cultured and exposed to cisplatin, carboplatin or vehicle for 24 h, followed by 24–240 h in culture or long-term xenografting. Survival, proliferation and apoptosis of prepubertal germ stem cell populations (gonocytes and spermatogonia), critical for sperm production in adulthood, were quantified. | Cisplatin, carboplatin (Platinum-based alkylating agent) | To determine the effect of clinically relevant exposure to platinum-based chemotherapeutics on human testis. | Survival, proliferation and apoptosis of prepubertal germ stem cell populations (gonocytes and spermatogonia), critical for sperm production in adulthood, were quantified. | 76 | 93 | 1 |
| 26 | Matilionyte et al., 2022b | *In vitro* | Human fetal (n = 23; 14–22 gestational weeks) testicular tissue pieces were exposed to cisplatin or vehicle for 24 h in vitro and analysed 24–240 h post-exposure or 12 weeks after xenografting. Human pre-pubertal (n = 10; 1–12 years) testicular tissue pieces were exposed to cisplatin (0.5 μg/ml), carboplatin (5 μg/ml) or vehicle for 24 h in vitro and analysed 24–240 h post-exposure. Immunohistochemistry was performed for SOX9 and anti-Müllerian hormone (AMH) expression and quantification was carried out to assess effects on Sertoli cell number and function respectively. AMH and inhibin B was measured in culture medium collected post-exposure to assess effects on Sertoli cell function. | Cisplatin, carboplatin (Platinum-based alkylating agent) | To investigate the effects of platinum-based chemotherapy exposure on somatic Sertoli cells in human foetal and pre-pubertal testis tissues. | Sertoli cell number and function. SOX9 and AMH protein expression, and apoptotic cells. | 76 | 93 | 1 |

^[[1]](#footnote-1)^

**Supplementary Table S2. Conversion of SciRAP score into reliability categories, based on information published by Wiklund and Beronius, 2022.**

| **SciRAP tool score conversion chart** | | |
| --- | --- | --- |
| Category | Description | Number of studies (n) |
| 1. Reliable | SciRAP methodological quality Score > 80 | 17 |
| 1. Reliable with restriction | SciRAP methodological quality Score > 65 | 7 |
| 1. Low reliability | SciRAP methodological quality Score <65 | 2 |

1. Risk of bias and quality were assessed using the appropriate (in vivo or in vitro) SciRAP (Science in Risk Assessment and Policy) scoring system, scirap.org providing a score, out of 100%, for methodological quality and reporting of results. *See Supplementary Table 2 for reliability groupings. ^#^Ray B, Potu BK. Ovarian folliculogenesis: detrimental effect of prenatal exposure to cyclophosphamide: a preliminary study. *Bratisl Lek Listy.* 2010; **111**: 369-372. [↑](#footnote-ref-1)
