# Supplementary material for: The impact of in utero exposure to cancer treatments on foetal reproductive development and future fertility: a systematic review
Source: Hum Reprod Open. 2025 Jul 23;2025(3):hoaf046. doi: 10.1093/hropen/hoaf046 (PMC12366489; doi:10.1093/hropen/hoaf046)
Supplement: hoaf046_Supplementary_Data [file hoaf046_supplementary_data.zip › Supplementary File S1.docx]

# Supplementary File S1. Search strategy

Key terms and databases:

Web of Science, google scholar and PubMed searches:

Combining 3 search queries:

[#1 OR #2 OR #3]

#1 (AB = ((‘Chemotherapy’ OR ‘anthracyclines’ OR ‘Topoisomerase inhibitors’ OR ‘alkylating

agents’ OR ‘antimetabolites’ OR ‘mitotic inhibitors’ OR ‘doxorubicin’ OR ‘epirubicin’ OR

‘etoposide’ OR 'VP-16' OR ‘busulfan’ OR ‘dacarbazine’ OR ‘cyclophosphamide’ OR

‘platinum’ OR ‘cisplatin’ OR ‘carboplatin’ OR ‘methotrexate’ OR ‘cytarabine’ OR ‘5-florouracil’

OR ‘vinca alkaloids’ OR ‘taxanes’ OR ‘paclitaxel’ OR ‘docetaxel’ OR ‘bleomycin’ OR

‘vinblastine’ OR ‘daunorubicin’ OR ‘temozolomide’ OR ‘mytomycin-C’ OR ‘antitumour

antitumo$r antibiotic’ OR ‘tamoxifen’ OR ‘ABVD’ OR ‘FAC’ OR ‘AC’) AND (‘exposure’ OR

‘administration’) AND (‘pregnancy’ OR ‘in utero’ OR ‘f$etal’ OR 'neonatal' OR 'maternal' OR

'prenatal') AND (‘fertility’ OR 'gonadal cell health' OR 'apoptosis' OR 'DNA damage' OR 'cell

death' OR ‘germ cell’ OR ‘sertoli’ OR ‘leydig’ OR ‘oocyte’ OR ‘testes’ OR ‘primordial follicles’

OR ‘granulosa’ OR ‘stromal’ OR ‘sex hormones’ OR ‘FSH’ OR ‘LH’ OR ‘oestrogen’ OR

‘testosterone’ OR ‘GnRH’ OR ‘menarche’ OR ‘sperm’ OR ‘reproduction’)))

#2 (TS = ((‘Chemotherapy’ OR ‘anthracyclines’ OR ‘Topoisomerase inhibitors’ OR ‘alkylating

agents’ OR ‘antimetabolites’ OR ‘mitotic inhibitors’ OR ‘doxorubicin’ OR ‘epirubicin’ OR

‘etoposide’ OR 'VP-16' OR ‘busulfan’ OR ‘dacarbazine’ OR ‘cyclophosphamide’ OR

‘platinum’ OR ‘cisplatin’ OR ‘carboplatin’ OR ‘methotrexate’ OR ‘cytarabine’ OR ‘5-florouracil’

OR ‘vinca alkaloids’ OR ‘taxanes’ OR ‘paclitaxel’ OR ‘docetaxel’ OR ‘bleomycin’ OR

‘vinblastine’ OR ‘daunorubicin’ OR ‘temozolomide’ OR ‘mytomycin-C’ OR ‘antitumour

antitumo$r antibiotic’ OR ‘tamoxifen’ OR ‘ABVD’ OR ‘FAC’ OR ‘AC’) AND (‘exposure’ OR

‘administration’) AND (‘pregnancy’ OR ‘in utero’ OR ‘f$etal’ OR 'neonatal' OR 'maternal' OR

'prenatal') AND (‘fertility’ OR 'gonadal cell health' OR 'apoptosis' OR 'DNA damage' OR 'cell

death' OR ‘germ cell’ OR ‘sertoli’ OR ‘leydig’ OR ‘oocyte’ OR ‘testes’ OR ‘primordial follicles’

OR ‘granulosa’ OR ‘stromal’ OR ‘sex hormones’ OR ‘FSH’ OR ‘LH’ OR ‘oestrogen’ OR

‘testosterone’ OR ‘GnRH’ OR ‘menarche’ OR ‘sperm’ OR ‘reproduction’)))

#3 (TI = ((‘Chemotherapy’ OR ‘anthracyclines’ OR ‘Topoisomerase inhibitors’ OR ‘alkylating

agents’ OR ‘antimetabolites’ OR ‘mitotic inhibitors’ OR ‘doxorubicin’ OR ‘epirubicin’ OR

‘etoposide’ OR 'VP-16' OR ‘busulfan’ OR ‘dacarbazine’ OR ‘cyclophosphamide’ OR

‘platinum’ OR ‘cisplatin’ OR ‘carboplatin’ OR ‘methotrexate’ OR ‘cytarabine’ OR ‘5-florouracil’

OR ‘vinca alkaloids’ OR ‘taxanes’ OR ‘paclitaxel’ OR ‘docetaxel’ OR ‘bleomycin’ OR

‘vinblastine’ OR ‘daunorubicin’ OR ‘temozolomide’ OR ‘mytomycin-C’ OR ‘antitumour

antitumo$r antibiotic’ OR ‘tamoxifen’ OR ‘ABVD’ OR ‘FAC’ OR ‘AC’) AND (‘exposure’ OR

‘administration’) AND (‘pregnancy’ OR ‘in utero’ OR ‘f$etal’ OR 'neonatal' OR 'maternal' OR

'prenatal') AND (‘fertility’ OR 'gonadal cell health' OR 'apoptosis' OR 'DNA damage' OR 'cell

death' OR ‘germ cell’ OR ‘sertoli’ OR ‘leydig’ OR ‘oocyte’ OR ‘testes’ OR ‘primordial follicles’

OR ‘granulosa’ OR ‘stromal’ OR ‘sex hormones’ OR ‘FSH’ OR ‘LH’ OR ‘oestrogen’ OR

‘testosterone’ OR ‘GnRH’ OR ‘menarche’ OR ‘sperm’ OR ‘reproduction’)))
